# Exploring the associations between lifestyle and dietary patterns with preclinical alzheimer’s disease: findings from La Rioja cohort study

**DOI:** 10.1007/s00394-026-04011-w

**Published:** 2026-06-06

**Authors:** María Iñiguez, Belinda Matute Tobías, Sara López-Álava, Silvia Yuste, Jose Ignacio Manzano, Emma Recio-Fernández, Patricia Pérez-Matute, Maria-Jose Motilva

**Affiliations:** 1 Infectious Diseases, Microbiota and Metabolism Unit, CSIC Associated Unit, Centre for Biomedical Research of La Rioja (CIBIR), C/Piqueras, 98, 26006 Logroño, La Rioja, Spain; 2 Neurology Service, Hospital Universitario San Pedro, C/Piqueras, 98, 26006 Logroño, La Rioja, Spain; 3 Instituto de Ciencias de la Vid y del Vino-ICVV (Consejo Superior de Investigaciones Científicas-CSIC, Universidad de La Rioja, Gobierno de La Rioja), Finca La Grajera, Ctra. de Burgos Km. 6 (LO-20, -salida 13), 26007 Logroño, La Rioja, Spain; 4 Lyfestyle, Microbiota and Health Unit, Health Sciences Faculty, University of La Rioja, C/Madre de Dios 53, 26006 Logroño, La Rioja, Spain

**Keywords:** Alzheimer’s disease, Cognitive assessments, Diet biomarkers, Dietary patterns, Lifestyle, Polyphenol urinary metabolites

## Abstract

**Purpose:**

Modifiable lifestyle factors play an important role in maintaining brain health. This study aimed to investigate lifestyle behaviours, with a special focus on dietary patterns, associated with preclinical Alzheimer’s disease, and to evaluate the urinary diet-polyphenol metabolites as objective biomarkers of plant-based food consumption by integration of the targeted metabolomics with self-reported dietary assessments.

**Methods:**

We conducted a case-control study including 50 individuals diagnosed with preclinical Alzheimer’s disease and 48 cognitively healthy controls (aged 55–75 years, both sexes). Demographic characteristics, cardiovascular risk factors, physical activity, cognitive and social engagement, and dietary patterns were assessed. The 24-hour urine samples were analysed by targeted ultra-high-performance liquid chromatography coupled to triple quadrupole tandem mass spectrometry (UHPLC–QqQ–MS/MS).

**Results:**

Dietary patterns characterised by higher daily consumption of vegetables and nuts and lower of processed foods, consistent with the MIND diet score, were associated with better cognitive outcomes. These associations were strengthened by engagement in cognitively stimulating activities. Urinary polyphenol metabolites, including anthocyanins, stilbenes, and gut microbiota-derived metabolites of dietary flavan-3-ols such as valerolactones and phenolic acids, were positively associated with the consumption of plant-based foods.

**Conclusion:**

Lifestyle modifications combining plant-rich dietary patterns with cognitive stimulation may support cognitive resilience in individuals with preclinical Alzheimer’s disease diagnosis. Urinary polyphenol metabolites represent objective biomarkers that can enhance the accuracy of diet-brain health assessments.

**Supplementary Information:**

The online version contains supplementary material available at 10.1007/s00394-026-04011-w.

## Introduction

According to the “Global Action Plan on the Public Health Response to Dementia 2017–2025” [1], dementia affected 55 million people worldwide in 2019, a figure that is predicted to increase to 139 million by 2050. Alzheimer’s disease (AD), the most common form of dementia, is a multifactorial neurodegenerative disease with distinct clinical stages, beginning with a long preclinical stage without cognitive impairments, and progressing to a prodromal stage characterised by mild cognitive impairment, followed by dementia [2]. AD does not exclusively affect older people, since young onset dementia (defined as symptom onset before the age of 65) accounts for up to 9% of cases. In sporadic AD, Aβ aggregates may begin to form approximately 20–30 years before clinical manifestation [3]. By the time the clinical manifestations become evident, typically before age 65 in early-onset cases and between 70 and 80 years in late-onset cases, a substantial pathological burden is already present [4].

During the preclinical stage of Alzheimer’s disease (AD), pathological brain changes, including abnormal amyloid-β (Aβ) deposition and tau pathology, are already present, although noticeable symptoms of cognitive decline or memory loss have not yet emerged [5]. Individuals remain cognitively normal in daily life, with no observable memory or behavioural impairments. Early clinical diagnosis relies on a combination of neuropsychological assessment, structural brain imaging techniques such as magnetic resonance imaging (MRI) or positron emission tomography (PET) [6–8], and established cerebrospinal fluid (CSF) biomarkers, including total tau, hyperphosphorylated tau, and the Aβ40/Aβ42 ratio [9, 10].

AD is influenced by both genetic predisposition and modifiable environmental factors, including diet, physical activity, and cognitive engagement [11]. Although most AD cases are sporadic, such genetic variants as the APOE gene located on chromosome 19q13, which encodes the protein that makes up numerous plasma lipoproteins, represent the strongest known genetic risk factor for AD, particularly in familial forms of the disease [12]. The APOE ε4 allele is associated with an earlier onset of AD, independently of other genetic factors [13]. This association is dose-dependent, so the carriers of two APOE ε4 copies tend to develop AD symptoms 10–15 years earlier than the average population [12].

While genetic predisposition and aging are key determinants of neurodegenerative disease, growing evidence suggests that modifiable lifestyle factors play an important role in maintaining brain health. Epidemiological studies consistently demonstrate that individual differences in AD risk are associated with adherence to a healthy lifestyle, including a balanced diet, regular physical activity, weight management, emotional resilience, and higher educational attainment [14, 15]. Among these factors, diet has emerged as a particularly important component in AD prevention and management. Adherence to the Mediterranean diet, characterised by high intake of fruit, vegetables, fish and whole grains, has been linked to slower memory decline and reduced medial temporal atrophy, potentially through mechanisms involving decreased amyloid-β and tau pathology [16, 17]. In contrast, Western dietary patterns are associated with increased inflammation, metabolic dysfunction, and impaired cerebral perfusion, all of which contribute to cognitive decline [18].

Social connectedness represents another key lifestyle factor associated with reduced dementia risk. Regular interactions with family and friends, active community participation, and living with others (including in intergenerational households), have been linked to healthier cognitive aging [19]. In addition, physical activity, particularly moderate-intensity exercise, together with sustained cognitive engagement through education, occupational attainment, and intellectually stimulating leisure activities, constitutes an essential non-pharmacological strategy for mitigating the risk of AD and slowing the progression of the disease [11].

Despite robust evidence supporting the influence of lifestyle factors on cognitive health, systematic research of the exposome in early-onset AD remains limited. In this case-control study, we examined lifestyle behaviours associated with dementia prevention in a cohort from the La Rioja region of Spain. The study included 50 individuals recently diagnosed with preclinical AD and 48 cognitively healthy controls, aged 55 to 75 years, from both sexes. We compared both groups in variables related to cardiovascular risk factors, physical activity, and cognitive and social engagement, with particular focus on dietary and nutritional patterns. Based on these comparisons, we further conducted correlation analyses to identify the lifestyle variables most strongly associated with preclinical AD. Additionally, to complement self-reported dietary data obtained from validated self-reported questionnaires, we quantified plant-derived polyphenol metabolites in 24-hour (24-h) urine samples as objective markers of dietary intake. The primary aim was to determine whether urinary metabolomic profiles were consistent with reported daily plant-food consumption and to evaluate the reliability of urinary phenolic metabolites as objective biomarkers of plan-food dietary exposure.

## Subjects and methods

### Participants

An observational case-control study was conducted. This included individuals with a recent diagnosis of preclinical AD (*n* = 50) and age- and sex-matched cognitively healthy controls (*n* = 48), aged 55–75. Participants were recruited between February 2021 and September 2022 through the Neurology Service of the Hospital Universitario San Pedro (Logroño, La Rioja, Spain). Patients with an AD diagnosis were enrolled via the *Dementia Early-Diagnosis Program* at the same hospital and subsequently invited to participate. Preclinical AD diagnosis followed the criteria of Ramusino et al. [20] and Younes et al. [21], integrating (1) neuropsychological evaluation, (2) CSF biomarkers, and (3) MRI measures.

Neuropsychological evaluation included the Mini-Mental State Examination (MMSE), a brief and simple screening tool assessing five cognitive domains (orientation, immediate memory, attention, delayed memory and language), with total scores ranging from 0–30 [22]. In addition, participants completed a comprehensive neuropsychological battery based on the Spanish Multicenter Normative Studies (NEURONORMA project) [23], widely used in clinical practice, which includes the Rey-Osterrieth Complex Figure (ROCF), and the Free and Cued Selective Reminding Test (FCSRT) tests. The ROCF assesses visual perception, constructional praxis and visuospatial memory, as well as higher-order cognitive abilities, such as planning and problem-solving strategies. The FCSRT evaluates verbal learning and memory. In accordance with the NEURONORMA standards, the scores from both tests were adjusted for age and educational level in all participants (both AD and control groups).

Inclusion criteria for the AD patients were: (1) MMSE > 24; (2) ROCF and FCSRT-IR results consistent with hippocampal memory impairment; (3) positive amyloid-β positron emission tomography (PET); and (4) CSF biomarker analysis with low Aβ42 levels, elevated total tau and phosphorylated tau (p-tau) and a reduced Aβ40/Aβ42 ratio. The cognitively healthy control group was selected with similar demographic characteristics to the AD participants as a whole. All control participants underwent the Cognitive Assessment Study to confirm normal cognitive function. For ethical reasons, the MRI and CSF analyses were not performed on the control group. In both groups, exclusion criteria included MMSE < 24 and major medical conditions, such as severe cerebrovascular disease, epilepsy or alcohol or drug abuse.

The study was approved by the Committee for Ethics in Drug Research in La Rioja, Spain (CEImLAR) (Reference CEImLAR P.I. 437). Personnel data were processed confidentially, respecting the basic ethical principles of research established by the Law 14/2007 on Biomedical Research and according to the Organic Law 15/1999 (concerning Data Protection), the Law 41/2002 (concerning Patient Autonomy) and the Law 14/1986 (concerning General Health). The Declaration of Helsinki, the Belmont Report and the UNESCO Universal Declaration on Bioethics and Human Rights were also followed. Contact with the participants was always carried out by authorised personnel and under medical supervision. All participants gave written informed consent to participate in this study and personnel data were processed confidentially. The collection and preparation of the biological samples (plasma and 24-h urine) was carried out by authorised personnel. Moreover, the biological samples were coded to maintain the confidentiality of the patient and, once analysed, were destroyed.

### Data collection

For both groups, demographic data collection and biological sampling were carried out across two visits scheduled one week apart (Supplementary Fig. S1). Visit 1 (V1): each participant was cited for an individual screening session during which a neuropsychologist explained the study, confirmed initial eligibility and obtained written informed consent. Participant provided demographic information, family history and medical history. Additionally, specific questionnaires were used to obtain data on leisure and social habits (Supplementary Material Questionnaire S1). Physical activity was assessed using the Physical Activity Classifier (Class AF), adapted from the ‘Guia de prescrició d’exercici físic per la salut (PEFS)’ of the Generalitat de Catalunya, Spain [24] (Supplementary Material Questionnaire S2). A fasting blood sample was collected, and each participant was provided with containers and detailed instructions for collecting a 24-h urine sample. Additionally, the participants received the document of the Three-Day Food Record (3-DFR) and were instructed to record all food and beverage intake over three consecutive days during the following week.

Visit (V2): Participants were cited to submit the 24-h urine sample and the fully completed 3-DFR, including photographs of each meal to enhance accuracy for size portion estimation. Dietary intake was further assessed during this visit using a validated semi-quantitative Food Frequency Questionnaire (FFQ), allowing for comprehensive evaluation of habitual dietary patterns.

### Dietary assessment and diet scores calculation

Dietary assessment was conducted using two questionnaires: the FFQ and the 3-DFR (Supplementary Fig. S1). The FFQ allows the long-term intake over the preceding year to be captured, thus accounting for seasonal variations as well as differences between weekdays and weekends. Participants were instructed to focus on habitual dietary habits rather than recent changes. This questionnaire, previously validated for the Spanish population [25], was adapted for this study by reducing the original 136 food items to 115, reflecting standard portion sizes commonly consumed by Spanish people (Supplementary Material Questionnaire S3). Specific questions regarding alcohol consumption and supplements were also included. The questionnaire was organised into nine food groups: I-Dairy foods; II-Eggs, meat, fish; III-Vegetables; IV-Fruits; V-Legumes and cereals, VI-Oils and fats; VII-Pastries, sweets; VIII-Miscellaneous; IX-Beverages. For each food, participants selected from nine frequency options: never or hardly ever; one to three times per month; once per week; two to four times per week; five to six times per week; once per day; two to three times per day; four to six times per day; and more than six times per day (Supplementary Material Questionnaire S3).

For accurate data collection, during Visit 2, information was obtained through a face-to-face interview conducted by two project trained researchers to complete the FFQ questionnaire. To ensure uniformity in data collection, all interviews (both AD and control) were conducted by the same trained personnel. For the AD group volunteers, the FFQ was completed with the cooperation of the patients’ parents or partners or relatives. The dietary intake pattern for each participant was expressed as the average daily consumption (in grams) of the nine food groups, calculated by summing the individual foods within each group.

Additionally, data obtained from specific items in the FFQ were used to calculate adherence scores for the MIND diet (partially based on the Mediterranean and DASH Intervention for Neurodegenerative Delay) [26] and the MEDAS diet (Mediterranean-Diet Adherence) [27]. The 15-point MIND diet score (Supplementary Material Table S4) comprises ten brain-healthy foods groups (green leafy vegetables, other vegetables, nuts, berries, beans, whole grains, seafood, poultry, olive oil and wine) and five unhealthy food groups (red meats, butter and stick margarine, cheese, pastries and sweets, and fried/fast food). Olive oil consumption was assigned a score of 1 if participants reported it as the primary oil usually used at home and 0 otherwise. For all other diet score components, we summed the frequency of consumption of each food item portion associated with that component, and a concordance score of 0, 0.5, or 1 was then assigned [26]. The MIND diet score was calculated as the sum of the 15 individual component scores (Supplementary Material Table S4).

The MEDAS consists of 12 questions on food consumption frequency and 2 questions on food intake habits considered characteristic of the Spanish Mediterranean diet [28] (Supplementary Material Table S5). Each question was scored 0 or 1. One point was given for using olive oil as the principal source of fat for cooking, preferring white meat over red meat, or for consuming: (1) 4 or more tablespoons (1 tablespoon = 13.5 g) of olive oil/day (including that used in frying, salads, meals eaten away from home, etc.); (2) 2 or more servings of vegetables/day; (3) 3 or more pieces of fruit/day; (4) 1 serving of red meat or sausages/day; (5) 1 serving of animal fat/day; (6) 1 cup (1 cup = 100 mL) of sugar-sweetened beverages/day; (7) 7 or more servings of red wine/wk; (8) 3 or more servings of pulses/wk; (9) 3 or more servings of fish/wk; (10) fewer than 2 commercial pastries/wk; 11) 3 or more servings of nuts/wk; or 12) 2 or more servings/wk of a dish with a traditional sauce of tomatoes, garlic, onion, or leeks sauteed in olive oil. If the condition was not met, 0 points were recorded for the category. The total MEDAS diet score was calculated by summing over all 14 of the component scores (Supplementary Material Table S5).

Daily intakes of energy, macro- and micronutrients were estimated for each volunteer using the 3-DFR questionnaire (Supplementary Material Questionnaire S6), an open-format tool that collects detailed information on all foods and beverages consumed over a specified period [29]. The questionnaire also includes information on food preparation methods, ingredients of mixed dishes and recipes. Participants were instructed to record all foods and beverages consumed over three days (of which one was a weekend day) during the week between Visits 1 and 2 (Supplementary Fig. S1). To improve the accuracy of portion size estimation, participants were encouraged to take photographs of their meals before and after consumption using their mobile phone camera. These photographs were sent to the person responsible in the study via MMS (multimedia message) or email. Based on the completed 3-DFR questionnaires and the photographs, the average daily intake of energy, macro- and micronutrients was calculated using the PCN Profood composition database [30]. Results were expressed as mean daily intakes of energy (kcal/day), macronutrients (g/day) and micronutrients (mg or µg/day).

### Assessment of genetic and clinical variables

Apolipoprotein E genotyping was performed using two TaqMan^®^ allele discrimination assays (C_3084793_20 and C_904973_10, Thermo Fisher Scientific, CA, USA) on a QuantStudio™ 5 Real-Time PCR System (Applied Biosystems, Foster City, CA, USA), following the manufacturer’s protocol. Genomic DNA for genotyping was isolated from frozen whole blood samples using the FlexiGene DNA kit (Qiagen) as previously described [31].

Body mass index (BMI; kg/m^2^) was calculated from measured body weight (kg) and height (m). Hypertension history was determined based on self-reported medical diagnosis, measured blood pressure (average of two measurements ≥ 160 mm Hg systolic or ≥ 90 mm Hg diastolic), or current use of antihypertensive medication. Diabetes history was determined based on self-reported medical diagnosis, fasting blood glucose levels (≥ 126 mg/dL), glycated hemoglobin (HbA1c) levels (≥ 6.5%) or current use of antidiabetic medication.

### Analysis of dietary polyphenol metabolites excreted in 24-h urine by targeted metabolomics (UHPLC/QqQ-MS/MS)

Dietary biomarkers were assessed through quantitative liquid chromatography analysis of urinary metabolites derived from dietary polyphenols excreted over a 24-hour period, following the method described by Jimenez-Salcedo et al. [32]. The retention time (RT) and multiple reaction monitoring (MRM) transitions used for the identification and quantification of each compound are provided in Supplementary Material Table S7. The quantification of some phenol metabolites was carried out using calibration curves generated from their corresponding commercial standards when available. The other compounds were tentatively quantified using the calibration curves of standards with similar chemical structures. The results were expressed as nmols (for anthocyanin metabolites) or µmols (for other phenolic metabolites) excreted in 24-h urine, taking into account the total urine volume collected over the 24-hour period for each participant.

### Statistical analyses

Categorical variables were presented as counts (percentages) and compared using the chi-square (χ²) test or Fisher’s exact test, as appropriate. Quantitative variables were expressed as mean ± standard deviation (SD) and compared using Student’s *t-*test. Covariate adjustment was performed using logistic regression analysis. Pearson’s correlation analysis was carried out to assess associations between continuous variables. All statistical analyses were performed using GraphPad Prism version 6 (GraphPad Prism^®^, La Jolla, California, USA), R-commander version 2.8-0, and R software version 4.3.1 (The R Foundation for Statistical Computing). Differences between the AD and control groups were considered statistically significant at *P <* 0.05.

## Results

### Characteristics of the study participants

The main demographic, clinical and lifestyle-related characteristics of the participants from the “La Rioja” cohort are summarised in Table 1. Women comprised 68% of the AD group and 62.5% of the control group. Significant differences between groups were observed for age, body mass index (BMI), APOE ε4 genotype distribution and neuropsychological scores. The age-adjusted MMSE score was significantly lower in the AD group (mean 26.67) than in the controls (mean 29.44). However, the mean MMSE values in both groups remained above the clinical cutoff of 24. Consistent with the MMSE results, neuropsychological assessments based on the Rey–Osterrieth Complex Figure (ROCF) and the Free and Cued Selective Reminding Test (FCSRT) revealed statistically significant group differences, with significantly lower performance observed in the AD group.

The distribution of the APOE ε4 genotype also showed significant differences between the two groups (*P <* 0.001). Specifically, the frequency of ε4 homozygotes (E4/E4) was higher in the AD group than in the controls (12 vs. 2.1%), as was the proportion of ε4 heterozygotes (E3/E4: 48 vs. 22.9%), whereas non-carriers were more prevalent among the controls (75 vs. 40%) (Table 1).

Regarding cardiovascular risk factors, no statistically significant differences were observed between the two groups in fasting blood glucose levels (adjusted for antidiabetic treatment) or total cholesterol concentration. Nevertheless, a higher proportion of individuals in the AD group exhibited fasting glucose levels above 100 mg/dL. The overall prevalence of comorbidities was similar between groups, with the exception of diabetes, which was significantly more frequent in the AD group (20%) compared with the controls (2.1%, *P =* 0.0128). Finally, educational attainment differed significantly between the groups (*P =* 0.0109), with a greater proportion of AD participants reporting basic education (50%) compared with the controls (25%) (Table 1).

**Table 1:** Basic characteristics, lifestyle and social habits, and physical activity of the study population

Characteristics	Control group	AD group	*P* value
Age (years), mean (SD)	64.7 (5.29)	70 (5.16)	**< 0.001**
Women, n (%)	30 (62.5%)	34 (68%)	0.567
BMI (kg/m^2^), mean (SD)	27.57 (4.16)	25.77 (3.45)	**0.022**
APOE ε4 genotype (n (%))			
*E2*,* E3*,* E3-E3 (non-carriers)*	36 (75%)	20 (40%)	**< 0.001**
*E3-E4 (heterozygote genotype)*	11 (22.9%)	24 (48%)	
*E4-E4 (homozygote genotype)*	1 (2.1%)	6 (12%)	
**Neuropsycological evaluation**			
MMSE score (SD)	29.44 (0.82)	26.67 (1.66)	< 0.001*
ROCF-Memory-Delayed recall raw score, mean (SD)	11.60 (2.49)	3.75 (1.93)	**< 0.001**
FCSRT-Total recall scaled score, mean (SD)	11.71 (2.61)	4.40 (3.36)	**< 0.001**
FCSRT-Delayed total recall scaled score, mean (SD)	13.35 (3.56)	3.78 (2.61)	**< 0.001**
**Cardiovascular risk factors**			
Fasting blood glucose (mg/dL), mean (SD)	96.44 (8.47)	100.76 (14.40)	0.252^**^
*Glucose < 100 mg/dL*,* (n(%))*	37 (77.1%)	26 (52%)	**0.035** ^**^
*Glucose 100–125 mg/dL*,* (n(%))*	10 (20.8%)	22 (44%)	
*Glucose > 126 mg/dL*,* (n(%))*	1 (2.1%)	2 (4%)	
Total cholesterol (mg/dL), mean (SD)	208.16 (43.77)	207.74 (30.88)	0.956
Total cholesterol > 200 mg/dL *(n(%))*	26 (54%)	26 (52%)	0.83
HDL-cholesterol (mg/dL), mean (SD)	65.26 (15.86)	62.51 (14.80)	0.376
LDL-cholesterol (mg/dL), mean (SD)	122.50 (40.45)	124.14 (27.75)	0.815
Systolic blood pressure (SBP), mean (SD)	141.12 (16.05)	144.58 (17.65)	0.314
*SBP > 130 mm Hg*,* (n (%))*	36 (75%)	26 (72%)	0.914
Diastolic blood pressure (DBP), mean (SD)	84.44 (11.65)	83.48 (11.20)	0.679
*DBP > 80 mm Hg*,* (n (%))*	28 (58%)	26 (52%)	0.669
Comorbidities			
*Diabetes (n(%))*	1(2.1%)	10 (20%)	**0.0128**
*Hypertension (n(%))*	13 (27.1%)	22 (56%)	0.0806
*Dyslipidemia (n(%))*	26 (54.2%)	23 (46%)	0.419
**Level of education**			
*Basic education*,* (n(%))*	12 (25%)	25 (50%)	**0.0109**
*Technical training*,* (n(%))*	7 (14.6%)	11 (22%)	
*High school*,* (n(%))*	11 (22.9%)	7 (14%)	
*Bachelor’s degree & Master’s degree (n(%))*	18 (37.5%)	7 (14%)	
**Lifestyle and social habits**			
Hours a day dedicated to watching TV (excluded weekend)			
*1 h or less*,* (n(%))*	9 (18.8%)	4 (8%)	**0.0497**
*Between 1–3 h*,* (n(%))*	29 (60.4%)	25 (50%)	
*More than 3 h*,* (n(%))*	10 (20.8%)	21 (42%)	
Hours a day dedicated to reading			
*1 h or less*,* (n(%))*	25 (52.1%)	41 (82%)	**0.0029**
*Between 1–3 h*,* (n(%))*	20 (41.7%)	9 (18%)	
*More than 3 h*,* (n(%))*	3 (6.2%)	0 (0%)	
Hours a day dedicated to memory activities			
*1 h or less*,* (n(%))*	30 (62.5%)	33 (66%)	0.934
*Between 1–3 h*,* (n(%))*	15 (31.3%)	14 (28%)	
*More than 3 h*,* (n(%))*	3 (6.3%)	3 (6%)	
Hours dedicated to leisure with friends or family			
*Weekends (hours)*,* (n(%))*	19 (39.6%)	11 (22%)	0.17
*Weekdays (hours)*,* (n(%))*	10 (20.8%)	13 (26%)	
*Every day of the week (hours)*,* (n(%))*	19 (39.6%)	26 (52%)	
Eating with company (family, friends, co-workers)			
*Eating alone*,* (n(%))*	11 (22.9%)	7 (14%)	0.171
*Occasionally eating with others*,* (n(%))*	4 (8.3%)	1 (2%)	
*Regularly eating with others*,* (n(%))*	33 (68.8%)	42 (84%)	
Hearing problems (n(%))	6 (12.5%)	11 (22%)	0.288
Hearing aid			
*No (n(%))*	46 (95.8%)	46 (92%)	0.807
*Sometimes (n(%))*	1 (2.1%)	1 (2%)	
*Yes (n(%))*	1 (2.1%)	3 (6%)	
**Physical activity**			
Physical activity at work or at home (n(%))	45 (93.8%)	49 (98%)	0.357
Physical exercise and/or sport (n(%))	42 (87.5%)	43 (86%)	1
Weekly frequency of physical exercise and/or sport			
*Never*,* (n(%))*	6 (12.5%)	7 (14%)	0.624
*1 or 2 times a week*,* (n(%))*	4 (8.3%)	4 (8%)	
*3 or more times a week*,* (n(%))*	14 (29.2%)	9 (18%)	
*Daily*,* (n(%))*	24 (50%)	30 (60%)	
Total score	6.10 (2.12)	6.14 (2.16)	0.934

Control group (*n* = 48), Alzheimer’s disease (AD) group (*n* = 50)

Mean (SD, standard deviation) was reported for continuous variables, while n (%) was used for binary variables. For the continuous variables, *t student* was used to determine whether there are any statistically significant differences between the means of control and AD groups. To examine the differences between categorical variables the chi-square test was used

Significant differences are indicated in bold and Legends in italics indicate binary variables, n(%)

*adjusted by age

**adjusted by pharmacological antidiabetic treatment

AD: Alzheimer’s disease, BMI: Body mass index, MMSE: Mini-Mental State Examination test

### Lifestyle and social habits

The main differences between the control and AD groups were observed in the number of hours that participants reported spending watching TV (*P =* 0.0497) and reading books (*P =* 0.0029) per day (Table 1). Specifically, 42% of participants in the AD reported watching TV for more than 3 h daily, compared with 20.8% in the control group. In contrast, 82% of individuals of the AD group reported reading for 1 h or less per day, whereas this percentage was 52.1% in the control group.

No statistically significant differences were detected between groups for other lifestyle variables, including time devoted to memory-related activities (e.g., crossword puzzles, word searches, card games or board games) or measures of social engagement, such as time spent in leisure activities with friends or family, eating in company (e.g., with family, friends, or co-workers), or the presence of self-reported hearing problems. Likewise, levels of physical activity did not differ significantly between the AD and control groups.

### Dietary patterns, and adherence to MIND and MEDAS diet scores

Table 2 summarises the dietary intake patterns of the study participants, calculated from the FFQ responses (Supplementary Material Questionnaire S3) and expressed as the average daily intake (g/day or mL/day) across nine food groups. Compared with the AD group, the control participants reported a significantly higher intake of vegetables (*P =* 0.0359) and a lower intake of the combined legumes and cereals category (*P =* 0.0407). Although the AD group showed a higher mean daily consumption of bakery and pastry products, this difference was not statistically significant. No significant differences were observed for the remaining food groups, likely reflecting the interindividual variability in dietary intake within each group. Further analysis of individual food items (Supplementary Table S8) revealed more specific differences between the two groups. The AD participants reported a significantly higher consumption of red meat (*P =* 0.0424) and industrial pastries (*P =* 0.00219), whereas the control participants reported a higher intake of nuts (*P =* 0.0174) and tea (*P =* 0.0256). No statistically significant differences were observed between groups in the daily consumption of the alcoholic beverages. Given that La Rioja is a traditional wine-producing region in which red wine is commonly consumed by elderly people, particular attention was paid to alcoholic beverage consumption patterns (Table 2). Based on FFQ data, participants were categorised in different levels of red wine consumption: non-consumers, low (< 100 mL/day), moderate (100–300 mL/day), or high (> 300 mL/day). A higher proportion of non-consumers was observed in the AD group (60.0%) compared with controls (33.3%), although no statistically significant differences were observed in overall red wine consumption patterns. Similarly, although a higher proportion of beer non-consumers was observed in the AD group, no significant differences were detected in beer consumption patterns between the groups (Table 2).

**Table 2:** Dietary intake pattern of study participants, based on the data of food frequency questionary (FFQ)

Food groups	Control group	AD group	*P* value
I. Dairy foods (g/day)	431.93 (261.98)	479.167 (237.56)	0.353
	[42.5–1168.0]	[115.53–1065.13]	
II. Eggs, meat, fish (g/day)	275.56 (72.57)	282.10 (74.50)	0.661
	[151.0–445.1]	[106.4–431.1]	
III. Vegetables (g/day)	740.99 (216.71)	637.93 (259.87)	**0.0359**
	[348.5–1187.0]	[141.1–1328.5]	
IV. Fruits (g/day)	279.45 (130.13)	276.18 (120.98)	0.898
	[61.6–894.7]	[24.2–598.5]	
V. Legumes & cereals (g/day)	225.33 (87.337)	269.78 (122.30)	**0.0407**
	[76.2–412.8]	[75.0–616.8]	
VI. Oils & fats (g/day)	68.59 (37.96)	60.23 (24.85)	0.203
	[19.1–228.5]	[23.5–150.8]	
VII. Bakery & Pastry (g/day)	54.46 (48.44)	72.81 (49.53)	0.0669
	[0.80–185.3]	[0.80–187.3]	
VIII. Miscellaneous	45.39 (44.16)	44.52 (33.20)	0.912
	[2.31–218.4]	[1.60–144.5]	
IX. Non-alcoholic beverages (mL/day)	1134.8 (351.8)	1167.2 (449.6)	0.691
	[557.0–1839.0]	[550.0–2639.0]	
IX. Alcoholic beverages (mL/day)	211.9 (252.72)	191.6 (264.72)	0.699
	[0.00–1075.0]	[0.00–1091.9]	
Red wine (mL/day)	*82.25 (120.2)*	*56.84 (103.8)*	*0.265*
*Non-consumers*,* (n(%))*	*16(33.3%)*	*30(60.0%)*	***0.0146***
Pattern of red wine consumption			
*Low (< 100 mL/day)*,* (n(%))*	*23(71.9%)*	*12(60.0%)*	
*Moderated (100–300 mL/day)*,* (n(%))*	*7(21.9%)*	*7(35.0%)*	*0.636*
*High (> 300 mL/d)*,* (n(%))*	*2(6.2%)*	*1(5.0%)*	
Beer (mL/day)	*102.0 (176.8)*	*104.6 (203.9)*	*0.947*
*Non-consumers*,* (n(%))*	*13(27.1%)*	*24(48.0%)*	***0.0388***
Pattern of beer consumption			
*Exporadic (< 330 mL/day)*,* (n(%))*	*31(64.6%)*	*20(40.0%)*	*0.300*
*Regular (> 330 mL/day)*,* (n(%))*	*4(8.3%)*	*6(12.0%)*	

Control group (*n* = 48), Alzheimer’s disease (AD) group (*n* = 50). See Supplementary Table S8 for full individual food data. Mean (standard deviation); [values range]

Mean (standard deviation) was reported for continuous variables, while n(%) was used for binary variables

For the continuous variables, *t* *student* was used to determine whether there are any statistically significant differences between the means of control and AD group

Significant differences are indicated in bold and Legends in italics indicate binary variables, n(%)

Adherence to the MIND diet, assessed using the corresponding adherence score (Supplementary Material Table S4), showed statistically significant differences between the groups (*P =* 0.0142), with higher adherence observed among the control participants (Table 3). When applying the predefined criterion for high adherence (≥ 10 points), a greater proportion of the control individuals met this threshold (47.9%) compared with the AD group (24%). Higher MIND diet adherence was characterised by greater consumption of berries and lower intake of red meat, pastries and sweets. In contrast, no significant differences between groups were observed for the MEDAS score, with the exception of a significantly lower consumption of pastries among the controls, consistent with the MIND diet findings. Overall adherence to the Mediterranean dietary pattern was relatively high in both groups, with 58% of the controls and 56% of the AD participants achieving MEDAS scores ≥ 9 (Table 3).

**Table 3:** MIND and MEDAS diet adherence scores, based on the data of FFQ. Control group (*n* = 48), Alzheimer’s disease (AD) group (*n* = 50)

Diet adherence score	Control group	AD group	*P* value
MIND diet Score (max 15 points)			
Score, mean (SD) [range]	9.73 (1.44) [5.5-12.5]	8.99 (1.46) [6.5-13.0]	**0.0142**
MIND diet adherence (≥10 points), (n(%))	23(47.9%)	12(24%)	**0.0135**
MIND diet score components (max 1 point)			
Green Leafy Vegetables^a^	0.72 (0.36)	0.60 (0.35)	
*≤ 2 servings/week, (n(%))*	*6(12.5%)*	*8(16%)*	0.142
*> 2 < 6/week, (n(%))*	*15(31.2%)*	*24(48%)*	
*≥ 6 servings/week, (n(%))*	*27(56.2%)*	*18(36%)*	
Other Vegetables^b^	0.75 (0.29)	0.75 (0.32)	
*< 5 serving/week, (n(%))*	*2(4.2%)*	*4(8%)*	0.615
*> 5- < 7 servings/week, (n(%))*	*20(41.7%)*	*17(34%)*	
*≥ 1 serving/day, (n(%))*	*26(54.2%)*	*29(58%)*	
Berries^c^	0.48 (0.36)	0.47 (0.47)	
*≤ 1 serving/week, (n(%))*	*13(27.1%)*	*23(46%)*	**0.00064**
*1 serving/week, (n(%))*	*24(50%)*	*7(14%)*	
*≥ 2 serving/week, (n(%))*	*11(22.9%)*	*20(40%)*	
Nuts^d^	0.54 (0.34)	0.47 (0.34)	
*≤ 1 serving/month, (n(%))*	*9(18.8%)*	*13(26%)*	0.612
*1/month < 5 week, (n(%))*	*26(54.2%)*	*27(54%)*	
*≥ 5 serving/week, (n(%))*	*13(27.1%)*	*10(20%)*	
Olive oil	1 (0)	1 (0)	
*Not primary oil, (n(%))*	*0(0%)*	*0(0%)*	1
*Primary oil used, (n(%))*	*48(100%)*	*50(100%)*	
Butter, Margarine	0.96 (0.14)	0.98 (0.10)	
*> 2 tablespoon/day, (n(%))*	*0(0%)*	*0(0%)*	
*1-2 tablespoon/day, (n(%))*	*4(8.3%)*	*2(4%)*	0.431
*< 1 tablespoon/day, (n(%))*	*44(91.7%)*	*48(96%)*	
Cheese	0.52 (0.31)	0.46 (0.28)	
*+7 serving/week, (n(%))*	*8(16.7%)*	*10(20%)*	
*1-6 serving/week, (n(%))*	*30(62.5%)*	*34(68%)*	0.487
*< 1 serving/week, (n(%))*	*10(20.8%)*	*6(12%)*	
Whole Grains	0.18 (0.3)	0.15 (0.27)	
*< 1 serving/day, (n(%))*	*34(70.8%)*	*37(74%)*	0.937
*1-2 serving/day, (n(%))*	*11(22.9%)*	*11(22%)*	
*≥ 3 serving/day, (n(%))*	*3(6.2%)*	*2(4%)*	
Fish (not fried)^e^	1 (0)	0.96 (0.14)	
*Rarely, (n(%))*	*0(0%)*	*0(0%)*	0.118
*1-3 serving/month, (n(%))*	*0(0%)*	*4(8%)*	
*≥ 1 servings/week, (n(%))*	*48(100%)*	*46(92%)*	
Beans^f^	0.6 (0.29)	0.6 (0.29)	
*< 1 serving/week, (n(%))*	*4(8.3%)*	*4(8%)*	1
*1-3 serving/week, (n(%))*	*30(62.5%)*	*32(64%)*	
*> 2 serving/week, (n(%))*	*14(29.2%)*	*14(28%)*	
Poultry (not fried)^g^	0.78 (0.37)	0.89 (0.29)	
*< 1 serving/week, (n(%))*	*7(14.6%)*	*4(8%)*	0.196
*1 serving/week, (n(%))*	*7(14.6%)*	*3(6%)*	
*≥ 2 serving/week, (n(%))*	*34(70.8%)*	*43(86%)*	
Red Meat and products^h^	0.39 (0.44)	0.30 (0.34)	
*+7 serving/week, (n(%))*	*25(52%)*	*25(50%)*	**0.0161**
*4-6 serving/week, (n(%))*	*9(18.8%)*	*20(40%)*	
*< 4 serving/week, (n(%))*	*14(29.2%)*	*5(10%)*	
Fast Fried Foods^i^	0.84 (0.29)	0.74 (0.31)	
*+4 serving/week, (n(%))*	*3(6.2%)*	*3(6%)*	0.0635
*1-3 serving/week, (n(%))*	*9(18.8%)*	*20(40%)*	
*< 1 serving/week, (n(%))*	*36(75%)*	*27(54%)*	
Pastries & Sweets^j^	0.58 (0.44)	0.35 (0.42)	
*+7 serving/week, (n(%))*	*15(31.2%)*	*27(54%)*	**0.0322**
*5-6 serving/week, (n(%))*	*10(20.8%)*	*11(22%)*	
*< 1 serving/week, (n(%))*	*23(47.9%)*	*12(24%)*	
Wine	0.39 (0.36)	0.27 (0.35)	
*> 2 glass/day or never, (n(%))*	*19(39.6%)*	*29(58%)*	0.22
*1 glass/month – 6 glass/week, (n(%))*	*21(43.8%)*	*15(30%)*	
*1 glass/day, (n(%))*	*8(16.7%)*	*6(12%)*	
**MEDAS diet Score (max 14 points)**			
Score, mean (SD) [range]	9.29 (1.72) [6-13]	8.88 (1.51) [5-12]	0.211
MEDAS diet adherence (≥9 points), (n(%))	28 (58%)	28 (56%)	0.977
MEDAS diet score components, (n(%))			
*Olive oil, main dressing*	48(100%)	49(98%)	1
*Olive oil, ≥ 4 tablespoons/day*	30(62.5%)	33(66%)	0.833
*Vegetables, ≥ 2 servings/day*	17(35.4%)	17(34%)	1
*Fruits, ≥ 3 servings/day*	23(47.9%)	18(36%)	0.306
*Read meat, <1 serving/day*	45(93.8%)	49(98%)	0.357
*Butter, <1 serving/day*	44(91.7%)	48(96%)	0.431
*Sweet beverages, <1 serving/day*	44(91.7%)	45(90%)	1
*Wine, ≥7 cups/week*	18(37.5%)	13(26%)	0.279
*Legumes, ≥ 3 servings/week*	22(45.8%)	31(62%)	0.156
*Fish and seafood, ≥ 3servings/week*	35(72.9%)	34(68%)	0.661
*Pastry, <3 servings/week*	23(47.9%)	13(26%)	**0.0357**
*Nuts, ≥ 3 servings/week*	30(62.5%)	25(50%)	0.229
*White meat preferred over red meat*	23(47.9%)	27(54%)	0.686
*Tomato soffritto*	44(91.7%)	42(84%)	0.357

For each individual food included in the MIND and MEDAS scores, respectively, values are assigned within a range of 0–1. n(%), n: number of volunteers (percentage of total volunteers in the group)

Mean (standard deviation) was reported for continuous variables, while n(%) was used for binary variables. For the continuous variables, *t student* was used to determine whether there are any statistically significant differences between the means of control and AD group

To examine the differences between categorical variables the chi-square test was used

Significant differences are indicated in bold and Legends in italics indicate binary variables, n(%)

^a^kale, collards, greens; spinach; lettuce/tossed salad

^b^green/red peppers, squash, cooked carrots, raw carrots, broccoli, celery, potatoes, peas, potatoes, tomatoes, string beans, beets, corn, zucchini, summer squash, eggplant, cucumber, coleslaw, cabbage, broccoli, onions, garlic, asparagus, artichokes, mushrooms

^c^strawberries

### Nutrient intake

Table 4 presents the mean daily intake of edible portion, energy and nutrients estimated from the 3-DFR questionnaire. No significant differences were observed between the groups in the total daily energy intake. In both groups, the mean energy intake was consistent with the European Food Safety Authority (EFSA) recommendations [33] for adults aged 60–70 years with a moderately active lifestyle (2293 kcal/day for men and 1863 kcal/day for women). Nevertheless, because the mean daily energy intake was slightly higher in the AD group, all subsequent nutrient analyses were adjusted for the total energy intake to ensure valid between-group comparisons. After energy adjustment, the AD group showed a significantly lower daily edible portion (*P* = 0.00334), a higher intake of total protein (*P =* 0.01270), primarily attributable to animal-derived protein (*P =* 0.0049), and a lower intake of plant-based protein (*P =* 0.0337). The daily intake of carbohydrates, dietary fibre, digestible polysaccharides and potassium was also significantly lower in the AD group. However, these differences were not statistically significant after adjustment for energy intake (Table 4). Regarding micronutrients, the participants in the AD group reported a lower daily intake of minerals and vitamins, with statistically significant differences observed for vitamin C (*P =* 0.0135) and folate (*P =* 0.0343). In addition, daily water intake was significantly lower in the AD group (*P =* 0.00164). No significant differences were observed in ethanol intake between groups, although the control group showed a higher mean daily ethanol consumption.

**Table 4:** Edible portion, energy and nutrient daily intake, based on the data of the three-day food record (3-DFR)

Nutrient	Control group	AD group	*P* value	Adjusted *P* value^a^
Edible portion (g/day)	16.63 (3.418)	14.11 (3.306)	**0.000351**	**0.00334**
Energy intake (kcal/day)	2024.8 (509.63)	1864.7 (340.16)	0.0721	
Total protein (g/day)	84.34 (23.70)	88.76 (29.44)	0.414	**0.01270**
*Animal protein (g/day)*	60.74 (18.62)	69.68 (29.34)	0.0743	**0.0049**
*Plant-based protein (g/day)*	23.59 (8.953)	19.08 (6.649)	**0.00588**	**0.0337**
Lipids (g/day)	108.6 (26.73)	100.7 (19.66)	0.0982	0.8470
*Saturated fatty acids (g/day)*	27.39 (8.972)	26.25 (7.698)	0.499	0.2350
*Monounsaturated fatty acids (g/day)*	53.47 (10.06)	49.99 (7.426)	0.0551	0.4070
*Polyunsaturated fatty acids (g/day)*	18.83 (9.489)	16.03 (6.786)	0.098	0.501
Cholesterol (mg/day)	377.1 (132.03)	388.3 (120.83)	0.663	0.2840
Total carbohydrates (g/day)	158.8 (52.05)	139.1 (37.92)	**0.0363**	0.272
Simple sugars/carbohydrates (g/day)	77.80 (26.45)	71.41 (27.05)	0.24	0.78
Dietary fiber (g/day)	21.17 (7.519)	17.90 (6.503)	**0.0237**	0.1130
Digestible polysaccharides (g/day)	81.07 (42.02)	67.73 (19.12)	**0.0486**	0.297
Sodium (mg/day)	2151.3 (832.7)	2029.3 (753.1)	0.449	0.4840
Potassium (mg/day)	3112.1 (749.7)	2751.1 (645.4)	**0.0124**	0.0872
Calcium (mg/day)	715.5 (214.3)	663.4 (196.9)	0.214	0.7530
Magnesium (mg/day)	308.2 (99.31)	270.1 (92.82)	0.053	0.3300
Phosphorus (mg/day)	1229.8 (321.5)	1204.9 (280.0)	0.684	0.1760
Iron (mg/day)	12.38 (3.778)	10.92 (3.791)	0.0591	0.2930
Zinc (mg/day)	8.617 (4.563)	7.917 (1.997)	0.332	0.1959
Vitamin A (µg/day)	1308.9 (2502.2)	817.6 (983.5)	0.209	0.2300
Retinoids (µg/day)	809.9 (2547.2)	402.4 (978.5)	0.304	0.2700
Vitamin B1 (Thiamine)(mg/day)	1.221 (0.410)	1.542 (1.120)	0.0624	**0.0166**
Vitamin B2 (Riboflavin(mg/day)	1.706 (0.592)	1.594 (0.460)	0.299	0.789
Vitamin B6 (mg/day)	1.889 (0.687)	1.787 (0.476)	0.395	0.8290
Vitamin B12 (µg/day)	8.236 (10.88)	6.165 (5.218)	0.237	0.2650
Vitamin C (mg/day)	146.9 (86.99)	104.1 (55.43)	**0.00498**	**0.0135**
Vitamin D (µg/day)	4.592 (3.940)	6.108 (7.009)	0.188	0.0663
Vitamin E (mg/day)	15.78 (6.826)	15.49 (8.815)	0.856	0.4470
Niacin (mg/day)	19.53 (6.309)	19.78 (6.134)	0.841	0.1410
Folate/Folic acid (µg/day)	290.6 (88.92)	241.2 (88.10)	**0.00687**	**0.0343**
Carotenoids (µg/day)	2993.2 (1483.5)	2494.3 (1974.9)	0.16	0.3320
Water (mL/day)	1407.9 (321.6)	1157.7 (290.9)	**0.000111**	**0.00164**
Ethanol (g/day)	10.05 (12.99)	6.123 (10.01)	0.0978	0.2980

Control group (*n* = 48), Alzheimer’s disease (AD) group (*n* = 50)

Mean (standard deviation)

^a^ Adjusted by energy intake

Significant differences are indicated in bold

### Dietary polyphenol intake and urinary biomarkers

Given the key dietary differences between the two groups, specifically, the higher daily intake of vegetables (Table 2) and plant-based proteins (Table 4) observed in the control group, a theoretical estimation of daily polyphenol intake was calculated for each participant. This estimation was based on the reported daily intake (g or mL/day) of plant-based foods obtained from the FFQ (Table 2), combined with the polyphenol content data from the Phenol-Explorer database [34]. Table 5 summarises the calculated daily polyphenol intake (mg/day), categorised by the main polyphenol classes and subclasses, including flavonoids, phenolic acids, stilbenes, lignans, phenyl alcohols and other minor phenolic compounds. Overall, no statistically significant differences were observed between the control and AD groups for the intake of the most abundant polyphenol classes. Nevertheless, total daily polyphenol intake, calculated as the sum of all quantified compounds, was higher in the control group (639.3 mg/day) than in the AD group (586.8 mg/day), which was consistent with the higher vegetable daily consumption reported by the controls (Table 2). Notably, a significantly higher intake of specific polyphenol subclasses was observed in the control group, these including dihydroflavonols (*P =* 0.0396), flavanols (*P =* 0.0339), hydroxybenzoic acids (*P =* 0.001) and stilbenes (*P =* 0.0105).

**Table 5:** Daily intake of plant-food polyphenols estimated from food frequency questionary (FFQ) data using the phenol explorer database, of the control (*n* = 48) and alzheimer’s disease (AD) (*n* = 50) groups

Plant-food polyphenols intake (mg/day)	Control group	AD group	*P* values^a^
Flavonoids (total)	284.1 (151.5)	239.6 (136.8)	0.13
Anthocyanins	38.32 (32.02)	30.33 (28.87)	0.198
Dihydrochalcones	5.523 (3.447)	6.544 (4.441)	0.206
Dihydroflavonols	2.023 (2.280)	1.096 (1.76)	**0.0396**
Flavanols	149.8 (93.73)	118.8 (78.76)	**0.0339**
Flavanones	20.51 (21.87)	22.29 (20.78)	0.681
Flavones	3.001 (3.118)	3.316 (3.109)	0.617
Flavonols	53.16 (19.09)	49.17 (17.90)	0.289
Isoflavonoids	1.879 (8.982)	6.489 (27.91)	0.272
Phenolic acids (total)	291.1 (155.1)	284.8 (138.3)	0.833
Hydroxybenzoic acids	29.84 (20.89)	17.73 (11.14)	**0.001**
Hydroxycinnamic acids	255.1 (145.8)	257.1 (142.3)	0.946
Hydroxyphenylacetic acids	1.256 (1.229)	1.370 (1.540)	0.687
Hydroxyphenylpropanoic acids	0.534 (0.619)	0.620 (0.781)	0.547
Stilbenes (total)	2.260 (2.700)	1.037 (1.420)	**0.0105**
Lignans (total)	4.952 (1.985)	4.924 (1.760)	0.941
Phenyl alcohols (tyrosol/hydroxytyrosol)(total)	40.66 (25.48)	41.85 (31.30)	0.836
Other (poly)phenols (total)	14.62 (2.362)	12.87 (2.555)	0.932
Alkylmethoxyphenols	1.144 (0.904)	1.241 (1.003)	0.614
Alkylphenols	6.415 (12.46)	5.82 (12.05)	0.811
Furanocoumarins	0.195 (0.343)	0.207 (0.483)	0.893
Hydroxybenzaldehydes	0.372 (0.310)	0.237 (0.270)	**0.0352**
Hydroxycoumarins	0.140 (0.130)	0.085 (0.130)	**0.0446**
Methoxyphenols	0.142 (0.110)	0.157 (0.133)	0.552
Naphtoquinones	1.752 (2.866)	1.084 (1.144)	0.138
Total ingested (poly)phenols (mg/day)	639.3 (274.3)	586.8 (209.0)	0.290

Mean (standard deviation)

^a^Significant differences are indicated in bold

To further investigate the potential of polyphenol metabolites as biomarkers of plant-based food intake, a targeted metabolic analysis of 24-h urine samples was conducted. This analysis aimed to determine whether urinary phenolic metabolite profiles reflected the dietary patterns derived from the FFQ data (Table 2). As shown in Table 6 and Supplementary Table S9, the average concentrations of a wide range of polyphenol metabolites, categorised by class and subclass, were quantified in 24-h urine samples. Although total urinary polyphenol excretion over 24 h was higher in the control group (981.4 µmol/24 h) compared with the AD group (881.1 µmol/24 h) (Table 6), this difference did not reach statistical significance. Among individual polyphenol classes, only total stilbene excretion was significantly higher in the control group (*P =* 0.0043), mainly due by increased levels of dihydroresveratrol (*P =* 0.0006) and its phase-II metabolite, dihydroresveratrol sulphate (*P =* 0.0007) (Supplementary Table S9). Notably, 24-hour urinary excretion of metabolites derived from all polyphenol classes was consistently higher in the control group, with several specific metabolites showing statistically significant differences (Supplementary Table S9).

**Table 6:** Dietary (poly)phenol metabolites excreted in 24-hour urine

Diet phenol metabolites excreted in 24-h urine	Control group	AD group	*P* values^a^
Total Anthocyanin metabolites (nmols/24 h)	19.78 (19.54)	18.21 (40.18)	0.806
Malvidin-3-glucuronide	7.550 (9.332)	4.287 (4.529)	**0.0291**
Cyanidin-3-arabinoside	0.142 (0.420)	0.014 (0.057)	**0.0364**
Peonidin-diglucuronide	0.173 (0.418)	0.038 (0.155)	**0.0359**
Total phenolic acids and metabolites (µmols/24 h)	480.26 (194.9)	440.5 (243.4)	0.373
*cis-*Coumaric acid	0.266 (0.162)	0.197 (0.176)	**0.0457**
*trans*-Coumaric acid	0.185 (0.241)	0.102 (0.064)	**0.0204**
2-Hydroxyphenylacetic acid	9.357 (8.312)	4.862 (6.306)	**0.0032**
4-Hydroxybenzoic acid	2.885 (1.731)	2.077 (1.234)	**0.0089**
Total proanthocyanidins and metabolites (µmols/24 h)	324.5 (360.8)	272.6 (256.8)	0.416
5-(3’,4’-dihydroxyphenyl)-valeric acid	0.675 (1.329)	0.225 (0.251)	**0.0207**
Total minor flavonoids (µmols/24 h)	0.098 (0.302)	0.142 (0.576)	0.6391
Total ellagitannin colonic metabolites (µmols/24 h)	2.552 (6.133)	1.764 (4.100)	0.459
Total phenyl alcohols and metabolites (µmols/24 h)	143.2 (84.44)	143.4 (200.2)	0.996
Total stilbenes and metabolites (µmols/24 h)	5.359 (5.594)	2.684 (2.444)	**0.0043**
Dihydroresveratrol	1.342 (1.511)	0.498 (0.460)	**0.0006**
Dihydroresveratrol sulphate	2.987 (3.302)	1.153 (1.036)	**0.0007**
Total lignans colonic metabolites (µmols/24 h)	8.782 (13.11)	6.516 (11.82)	0.372
Sum Catechols, pyrogallols and phloroglucinols (µmols/24 h)	16.54 (11.15)	14.01 (10.27)	0.246
Total (poly)phenols metabolites excreted (µmols/24 h)	981.4 (488.2)	881.1 (565.5)	0.283

Results are presented as the sum of total metabolites excreted for each phenolic group, as well as the daily excretion levels of individual metabolites that showed statistically significant differences between the two groups (control, *n* = 48; AD, *n* = 50)

Mean (standard deviation)

^a^Significant differences are indicated in bold

### Correlation analysis of cognitive status, lifestyle, dietary patterns and urinary biomarkers

**Fig. 1:**
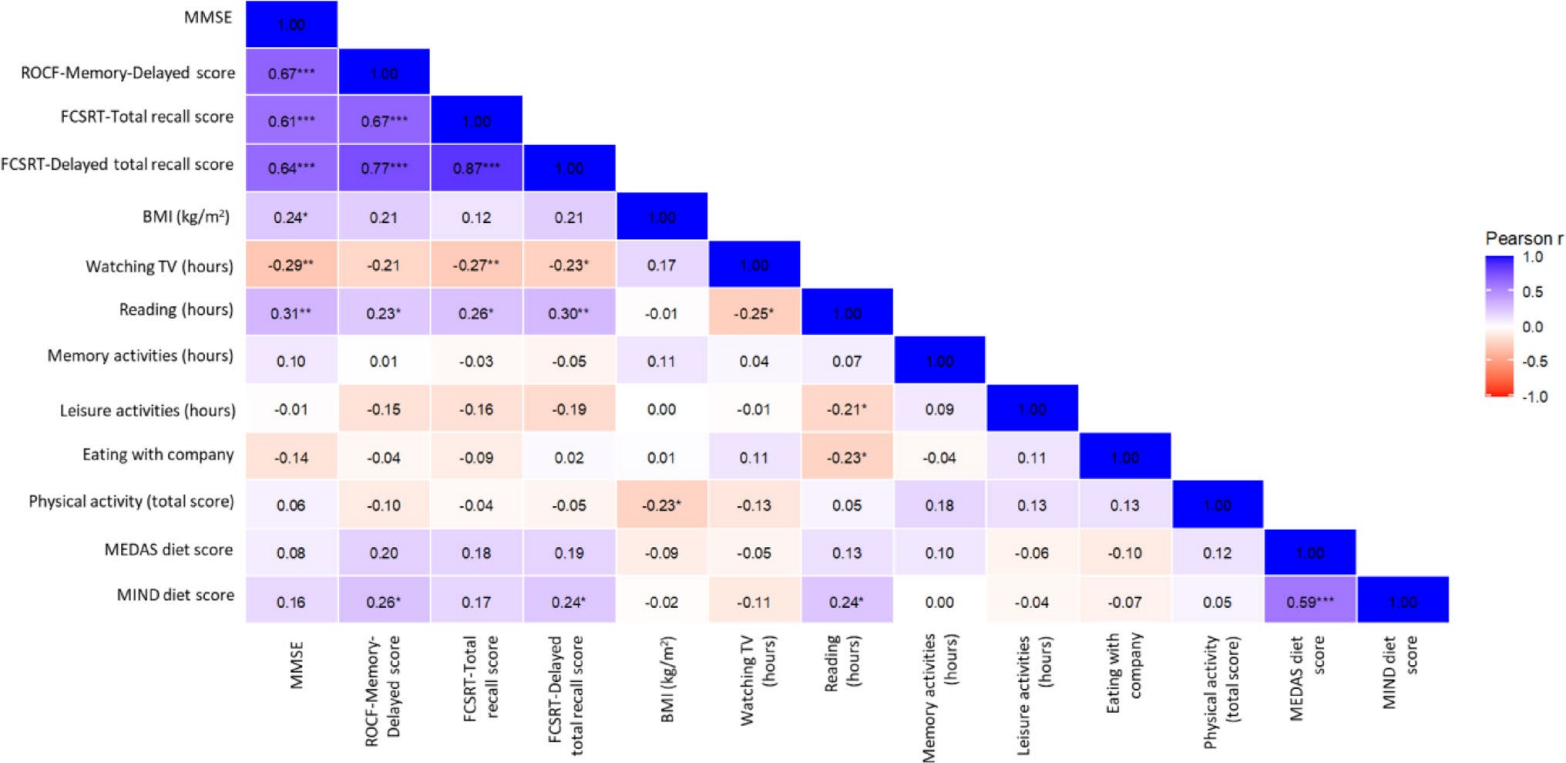
Correlation matrix of characteristics, lifestyle factors and social habits in the La Rioja cohort. * *P <* 0.05, ** *P <* 0.01, *** *P <* 0.001

To explore the relationships among cognitive status, lifestyle behaviour and dietary patterns, correlation analyses were performed using data from both the control and AD participants (Fig. 1). Variables included the neuropsychological scores (MMSE, ROCF and FCSRT), lifestyle and social habits (Table 1), and adherence scores for healthy dietary patterns (MIND and MEDAS) (Table 3). Notably, neuropsychological scores were positively correlated with time spent reading per day and negatively correlated with time spent watching television (Fig. 1). In addition, a significant and negative correlation was observed between time spent reading and time watching TV (*P <* 0.05). The significant positive association between the MIND diet score and ROCF and FCSRT scores (*P <* 0.05) is interesting, indicating that healthier cognitive and lifestyle behaviours clustered with healthier dietary patterns.

**Fig. 2:**
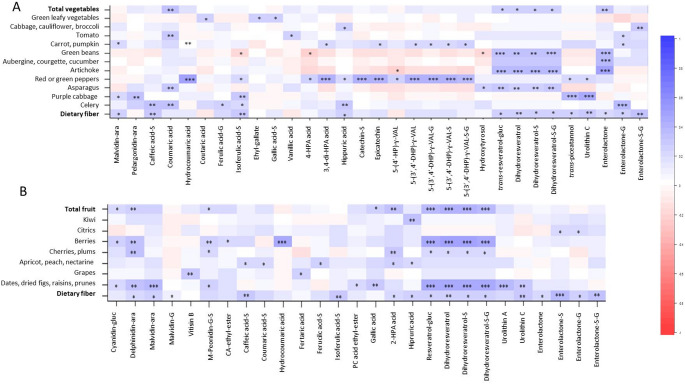
Heatmap of the main correlation coefficients between daily intake of vegetables (**A**) and fruits (**B**) and polyphenol metabolites quantified in 24-h urine samples. Colour intensity reflects the strength and direction of the associations, with blue indicating positive correlations and red indicating negative correlations. * *P <* 0.05, ** *P <* 0.01, *** *P <* 0.001. gluc: glucoside; ara: arabinoside; G: glucuronide; S: sulphate; M: methyl; CA: caffeic acid; PC: protocatechuic; HP: hydroxyphenyl; DHP: hydroxyphenyl; HPA: hydroxyphenylacetic; VAL: valerolactone

**Fig. 3:**
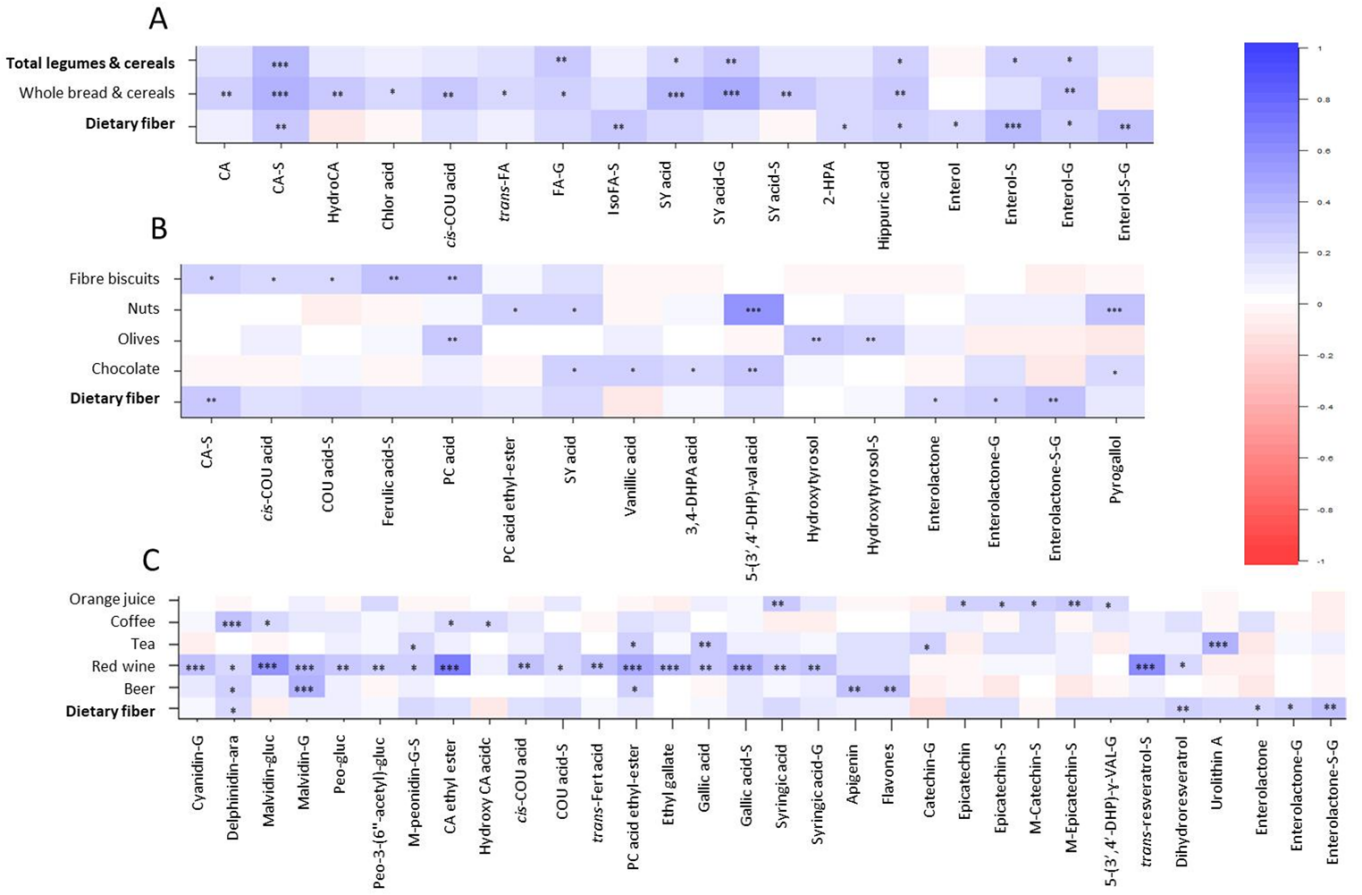
Heatmap of the main correlation coefficients between daily intake of legumes & cereals (**A**), various foods (**B**) and beverages (**C**) and polyphenol metabolites quantified in 24-h urine samples. Colour intensity reflects the strength and direction of the associations, with blue indicating positive correlations and red indicating negative correlations. * *P <* 0.05, ** *P <* 0.01, *** *P <* 0.001. gluc: glucoside; ara: arabinoside; G: glucuronide; S: sulphate; M: methyl; CA: caffeic acid; PC: protocatechuic; HP: hydroxyphenyl; HPA: hydroxyphenylacetic, DHP: dihydroxyphenyl; DHPA: dihydroxyphenylacetic; val: valeric acid; VAL: valerolactone; Chlor: Chlorogenic acid; COU: coumaric acid; SY: syringic acid; Enterol: Enterolactone; Peo: Peonidin-3-(6’’-acetyl)-glucoside, Fert: Fertaric acid

Next, potential biomarkers of plant-based food intake were explored by examining correlations between 24-h urinary polyphenol metabolites and the daily consumption of plant-derived foods estimated from the FFQ data. The most relevant correlations are shown in Figs. 2 and 3. Total vegetable intake was significantly correlated (*P* < 0.01) with urinary excretion of coumaric acid and enterolactone (Fig. 2A), a mammalian lignan produced by the gut microbial metabolism of dietary lignans [35]. At the individual vegetable level, intake of green beans and artichokes was associated with higher urinary concentrations of stilbenes, specifically resveratrol derivatives (including phase-II metabolites) and enterolactone. Moreover, consumption of red and green peppers, frequently consumed in the La Rioja region, was significantly correlated with urinary valerolactones, microbial metabolites derived from dietary catechins and epicatechins [36].

Total fruit intake was significantly associated with urinary excretion of the anthocyanins delphinidin and cyanidin (Fig. 2B), reflecting the consumption of berries, cherries and dried fruits. In addition, positive correlations (*P <* 0.001) were observed between total fruit intake and urinary concentrations of resveratrol-derived metabolites and urolithins, microbial products of ellagitannin colonic fermentation [37], particularly driven by the consumption of berries and dried fruits (Fig. 2B).

Analysis of other plant-based foods and beverages revealed additional diet-biomarker relationships (Fig. 3). Intake of legumes and cereals, particularly wholemeal bread and wholegrain rice and pasta, was positively correlated with urinary phenolic acids, including caffeic acid sulphate and syringic acid, detected both as free compounds and as phase-II hepatic metabolites (sulphated and glucuronidated) (Fig. 3A). Nut consumption was associated with higher urinary levels of valeric acid derivatives, microbial metabolites of catechin and epicatechin, as well as pyrogallol, likely derived from such pyrogallol-containing polyphenols as epigallocatechin gallate and tannic acid [38]. Correlation analysis of daily beverage intake (Fig. 3C) revealed a strong association (*P <* 0.001) between red wine consumption and urinary excretion of several anthocyanins, including native compounds, such as malvidin-glucoside, and phase-II hepatic metabolites, such as cyanidin-glucuronide and malvidin-glucuronide. Significant positive correlations were also observed between red wine consumption and urinary concentrations of phenolic acids, gallic acid derivatives and the phase-II metabolite resveratrol-sulphate.

**Fig. 4:**
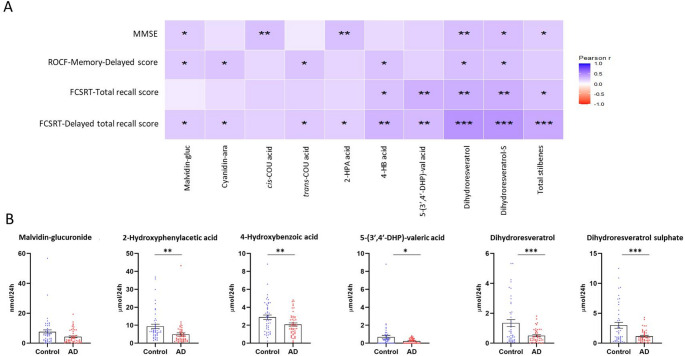
Heatmap of the main correlation coefficients between cognitive scores and most relevant polyphenol metabolites detected in 24-h urine (**A**); urinary excretion quantified in 24-h urine samples of the most relevant polyphenol metabolites within the control and AD groups (**B**). *P < 0.05, ** *P <* 0.01, *** *P <* 0.001. ara: arabinoside; G: glucuronide; S: sulphate; COU: coumaric acid; HPA: hydroxyphenylacetic; HB: hydroxybenzoic; DHP-val: dihydroxyphenyl valeric acid

Finally, associations between urinary polyphenol metabolites and cognitive performance across neuropsychological domains were analysed (Fig. 4). Significant positive correlations were observed between neuropsychological scores, including MMSE, ROCF, and FCSRT, and several key urinary metabolites (Fig. 4A). Total stilbenes, two resveratrol derivatives and 5-(3′,4′-dihydroxyphenyl)-valeric acid showed the strongest correlations across all cognitive scores. Significant correlations were detected for some simple phenolic acids and for the anthocyanins malvidin-3-glucuronide and cyanidin-3-arabinoside. The metabolites showing the strongest correlations were quantified at higher concentrations in the 24-h urine of the control group (Fig. 4B).

## Discussion

Preclinical AD is characterised by the presence of amyloid-β (Aβ) pathology, with or without tau pathology, in the absence of clinically overt cognitive impairment. Within the “La Rioja” cohort, individuals in the AD group were, on average, older than the controls. However, age-adjusted MMSE scores showed only modest reductions that remained within the normal range, supporting a preclinical rather than a symptomatic disease stage. This clinical classification was further corroborated by performance on sensitive neuropsychological measures, adjusted for age and education, including the Rey–Osterrieth Complex Figure (ROCF) and the Free and Cued Selective Reminding Test (FCSRT), which revealed subtle impairments in visuospatial, executive and episodic memory functions, domains known to be affected early in AD [23]. Consistent with previous findings, these results indicate that domain-specific neuropsychological assessments can detect early cognitive changes despite preserved global cognitive performance [39], supporting the presence of subtle inefficiencies alongside intact everyday functioning in preclinical AD and underscoring the value of comprehensive cognitive assessment for early disease characterisation.

Regarding cardiovascular risk factors, no major differences were observed between the control and AD groups, except for glucose metabolism. Higher fasting blood glucose levels and greater prevalence of diabetes in the AD group support evidence that metabolic dysfunction contributes to dementia risk [40], potentially interacting with other pathological processes in early AD. Genetic analysis further highlighted a significantly higher frequency of the APOE ε4 allele among AD individuals, consistent with its role in impaired amyloid clearance, neuroinflammation, and blood–brain barrier dysfunction [41–43], whereas the ε2 and ε3 alleles may confer relative protection [44]. These findings highlight the interplay between genetic susceptibility and metabolic risk in modulating AD disease vulnerability.

Beyond these inherent factors, modifiable lifestyle determinants, particularly cognitive reserve (CR), remain critical. Lower educational attainment in the AD group aligns with evidence linking higher education and other lifelong cognitively stimulating experiences, including occupational complexity, to reduced dementia risk through enhanced neural efficiency and synaptic density, enabling the brain to better compensate for neuropathology and explaining why similar pathological burdens may yield divergent clinical outcomes [45–47]. Notably, CR appears to delay the clinical onset of cognitive impairment rather than modifying the rate of decline once symptoms emerge [48], emphasising the importance of early- and mid-life interventions.

Cognitively, socially and physically engaging leisure activities support CR through mechanisms such as enhanced neurogenesis, vascular health, stress reduction and social support [49]. In our cohort, the AD participants spent more time watching TV and less time reading than the controls, and neurocognitive performance (MMSE, ROCF, FCSRT) was negatively associated with TV time and positively with reading. Consistent with longitudinal evidence that regular reading lowers later-life cognitive decline, independently of education [50]. Other lifestyle factors, including physical activity, social engagement, and memory-based leisure activities, did not differ significantly between the two groups, likely reflecting the relatively homogeneous sociocultural context of the “La Rioja” cohort. These findings underscore the potential of cognitively stimulating activities as a feasible, low-cost strategy to delay the clinical manifestation of AD, even among individuals with elevated genetic or metabolic risk.

Dietary patterns are increasingly recognised as modulators of cognitive health through their effects on inflammation, oxidative stress, vascular function and neuropathological burden [51]. In our cohort, overall adherence to the Mediterranean diet was largely homogeneous between groups, reflecting shared cultural dietary habits. However, meaningful differences emerged in specific food choices. Individuals in the AD group reported a lower intake of vegetables and a higher consumption of refined cereals, pastries, desserts, red meat and ultra-processed foods, whereas the controls consumed more nuts and traditional plant-based foods, such as “gazpacho”. These patterns are consistent with extensive evidence linking higher consumption of minimally processed plant foods to reduced risk of cognitive decline and neurodegenerative diseases [52], partly mediated by such bioactive compounds as polyphenols, which modulate neuroinflammation, support vascular function and promote neurogenesis [53]. Beverage consumption also differed between groups, with the control group reporting a higher intake of tea, coffee and red wine. Although total alcohol consumption was comparable, moderate red wine intake with meals was more prevalent among the controls, consistent with evidence that wine consumption within a Mediterranean dietary context may confer cognitive and cardiometabolic benefits through antioxidant and anti-inflammatory mechanisms, effects not observed with excessive alcohol intake [54, 55].

Although overall Mediterranean diet adherence (MEDAS) did not differ significantly between the groups, adherence to the MIND diet, a pattern specifically designed to promote neuroprotection, was higher among the controls. This difference was driven by greater intake of leafy green vegetables, berries and nuts, dietary components repeatedly associated with cognitive resilience and reduced AD risk, and lower consumption of red meat and fried or sugary foods [26, 56, 57]. The slightly stronger association observed for the MIND diet may reflect its emphasis on foods rich in neuroprotective nutrients, including antioxidants and fibre [58].

In line with this pattern-based observations, the controls exhibited a more favourable nutritional profile, characterised by a higher intake of plant-based proteins, dietary fibre and key micronutrients, particularly vitamin C and folates. Vitamin C plays a critical role in mitigating oxidative stress, supporting neuronal development and modulating neurotransmitter synthesis [59], while folate is essential for homocysteine metabolism, DNA synthesis, methylation processes and neurotransmitter production, pathways implicated in cognitive function and AD pathogenesis [60]. Collectively, these findings suggest that subtle but consistent differences in diet quality, rather than overall dietary adherence, may contribute to cognitive resilience in this population.

Beyond micronutrients, increasing evidence underscores the importance of plant-derived bioactive compounds, particularly polyphenols, as key mediators of diet-related neuroprotection. Polyphenols exert potent antioxidant and anti-inflammatory effects and modulate multiple AD related pathways, including amyloid aggregation, synaptic dysfunction, neuroinflammation, oxidative stress, cerebrovascular impairment and protein clearance [61]. Importantly, their extensive gastrointestinal and gut microbial metabolism generates bioactive metabolites that modulate inflammation, shape gut microbial ecology and contribute to gut-brain axis communication, making qualitative differences in intake potentially relevant for neuroprotective effects [62, 63]. In the present study, although total polyphenol intake did not differ significantly between the groups, cognitively healthy controls exhibited a higher overall intake and significantly greater consumption of specific polyphenol classes, including flavanols, hydroxybenzoic acids and stilbenes.

The complex metabolic fate of polyphenols generates a wide range of metabolites that can serve as biomarkers of dietary exposure, overcoming inherent limitations of such self-reported dietary assessment tools as FFQs and 3-DFR. In this context, the NIH 2020–2030 Strategic Plan for Nutrition Research [64] emphasises the integration of metabolomic approaches to advance precision nutrition and capture interindividual variability in dietary responses. Urine represents an optimal biofluid for metabolomic studies due to its non-invasive collection, high participant compliance and rich metabolite composition reflecting diet, metabolism and health status [36, 65]. In our study, most individual polyphenol-derived metabolites were detected at higher concentrations in the controls, likely reflecting their greater intake of polyphenol-rich plant foods. Most urinary polyphenol-derived metabolites detected in this study consisted of phase-II hepatic conjugates, primarily glucuronides and sulphates, along with a broad range of low-molecular-weight phenolic acids in both free and conjugated forms. These profiles are consistent with extensive host and gut microbial metabolism of dietary polyphenols. Correlation analyses between FFQ-derived plant food intake and 24-h urinary metabolites further supported the higher intake observed in the control group. These findings align with previous metabolomic studies identifying valerolactones and benzoic acid derivatives as robust markers of long-term exposure to flavonoid-rich diets [36]. In our cohort, valerolactones and phenolic acids, derived from extensive gut microbial metabolism of proanthocyanidins and flavanols [66], were the predominant urinary polyphenol metabolites, with consistently higher concentrations in the controls, although group differences did not reach statistical significance. Notably, proanthocyanidin intake has been associated with preserving brain metabolic activity in older adults with mild cognitive impairment [67], supporting the biological relevance of the higher metabolite levels observed in the control group. Similarly, urinary resveratrol metabolites, including sulphate and glucuronide conjugates, were more abundant in the control group and correlated positively with the intake of vegetables commonly consumed in the La Rioja population (e.g., green beans, artichokes and asparagus). Urinary enterolactone derivatives, microbial metabolites of dietary lignans, were also positively associated with vegetable intake. As reliable biomarkers of lignan exposure [68], enterolignans exert antioxidant, anti-amyloid, mitochondrial, and anti-inflammatory effects that may contribute to cognitive resilience [69].

Regarding fruit consumption, urinary resveratrol metabolites emerged as potential markers of total daily fruit consumption, particularly berries, raisins and prunes, consistent with the higher concentration of these metabolites in the 24-h urine of control group. In addition, specific urinary anthocyanins were positively associated with red fruit intake. Although the total urinary anthocyanin concentration did not differ significantly between the groups, several individual anthocyanins were significantly higher in the control group. This observation is biologically relevant given evidence that anthocyanins and their metabolites can cross the blood-brain barrier and promote cognitive function through neurogenesis modulation, synaptic plasticity, mitochondrial function, oxidative stress and neuroinflammation [70].

Consumption of legumes and cereals was positively correlated with the urinary excretion of simple phenolic acids (e.g., caffeic, chlorogenic, ferulic and syringic acids) and their hepatic conjugates, supporting their utility as biomarkers of whole-grain and legume intake. Enterolactone and its phase-II conjugates were also associated with these food groups, consistent with whole grains and seeds being major dietary lignan sources [69]. Additionally, 5-(3′,4′-dihydroxyphenyl) valeric acid, a gut microbiota-derived metabolite of proanthocyanidins, emerged as a potential biomarker of nut and chocolate consumption. The higher levels of this metabolite observed in the control group are noteworthy, given its reported capacity to restore synaptic plasticity and reduce amyloid-β burden in experimental models [66].

Olive consumption was positively associated with urinary hydroxytyrosol and its sulphate conjugate, confirming their usability as specific biomarkers of olive-derived phenolic intake. Although hydroxytyrosol can also be produced endogenously via dopamine metabolism, it is widely recognised for its neuroprotective properties, including attenuation of amyloid-β deposition, tau hyperphosphorylation, oxidative stress and neuroinflammation [71, 72]. The absence of significant differences in urinary concentrations between the AD and control groups likely reflects the similar consumption of virgin olive oil in both groups, supporting their role as dietary intake markers rather than disease-related indicators.

Given the established health benefits of dietary fibre and its role as a carrier of polyphenols, daily fibre intake assessed by the 3-DFR was included in the correlation analysis. Fibre intake was positively associated with several urinary polyphenol-derived metabolites, including resveratrol derivatives, enterodiol and enterolactone. Fibre-polyphenol complexes enhance colonic delivery of bound polyphenols, promoting microbial fermentation, short-chain fatty acid production and gut microbiota diversity [73, 74]. Although fibre intake did not differ significantly between the groups, the trend toward higher intake in the control group, together with observed metabolite patterns, highlights the close interplay between dietary fibre, polyphenol metabolism and microbial activity.

Among beverages, red wine consumption showed the strongest associations with urinary polyphenol metabolites, including anthocyanins, phenolic acids and stilbenes. Malvidin-glucoside, caffeic acid ethyl ester and *trans-*resveratrol sulphate emerged as particularly robust biomarkers, consistent with previous targeted metabolomic studies identifying stilbenes as highly specific intake markers despite their relatively low abundance in wine [32]. Tea consumption was positively associated with urinary urolithin A, a gut microbiota-derived metabolite of ellagitannins. Although group differences were not statistically significant, total urolithin excretion was higher in the controls. This finding is of interest given evidence that urolithin A crosses the blood–brain barrier and has been associated with reduced neuroinflammation, enhanced neurogenesis and lower age-related brain atrophy, particularly in the hippocampus [75]. Overall, urinary phenolic metabolites provided objective support for the dietary differences observed between the AD and control groups and complemented self-reported dietary data by reducing recall bias. Although only a subset of metabolites differed significantly between groups, this variability likely reflects differences in intake levels, bioavailability, gut microbiota composition and interindividual metabolic responses rather than a lack of biological relevance.

Interestingly, urinary polyphenol metabolites were associated with cognitive performance. Total stilbenes and resveratrol-derived metabolites were positively correlated with neuropsychological scores, with the strongest correlations observed for verbal learning and memory (FCSRT), alongside additional associations with visuospatial and executive functions (ROCF) and global cognitive status (MMSE). These findings align with evidence suggesting that long-term dietary exposure to resveratrol may support cognitive function, particularly memory-related processes, potentially through effects on hippocampal connectivity, cerebrovascular regulation and synaptic plasticity [76, 77]. Notably, cognitive benefits appear more pronounced when resveratrol is consumed within whole-food matrices, such as grape-derived products and wine, suggesting synergistic interactions with other polyphenols that enhance bioavailability or biological efficacy [52].

It was of particular interest that FCSRT performance was positively associated with microbial-derived flavan-3-ol metabolites, including 5-(3′,4′-dihydroxyphenyl)valeric acid and several simple phenolic acids. Flavan-3-ols undergo extensive colonic metabolism, yielding phenyl-γ-valerolactones and subsequently valeric acid derivatives, along with a range of low-molecular-weight phenolic acids [78, 79]. Evidence from neuronal cell models supports the neuroprotective potential of these metabolites, suggesting roles in neurite outgrowth and attenuation of oxidative stress [80, 81]. Our findings support the hypothesis that the cognitive benefits associated with flavan-3-ol intake may be primarily mediated by their microbial-derived metabolites rather than by native compounds, although direct evidence in humans remains limited.

Finally, positive associations were observed between anthocyanins, particularly malvidin-glucuronide and cyanidin-arabinoside, and cognitive performance. These findings are consistent with intervention studies reporting anthocyanin-related benefits across multiple cognitive domains, including global cognition, episodic and working memory, visuospatial processing, attention and psychomotor speed [70]. These studies have revealed stronger effects following consumption of anthocyanins from whole foods rather than supplements, and with longer intervention durations, independently of age.

Taken together, our findings support the use of stilbenes as biomarkers of fruit, vegetable and red wine intake, valerolactones as markers of flavan-3-ol–rich foods such as nuts and chocolate; and anthocyanins as indicators of red fruit and wine consumption. Notably, the observed associations between specific gut microbiota-derived metabolites and memory performance suggest that the cognitive effects attributed to dietary polyphenols may be mediated, at least in part, by microbiota–derived metabolites rather than by native dietary compounds present in foods. Although causal inferences cannot be drawn, our results contribute to growing evidence that diet-microbiota interactions represent modifiable determinants of cognitive health and highlight the need for well-controlled intervention studies to clarify their mechanistic roles in neuroprotection and the prevention of cognitive decline.

### Strengths and limitations

This study has several strengths that enhance its scientific rigour and translational relevance. First, the rigorous clinical characterisation of the “La Rioja” cohort was conducted by a specialised multidisciplinary team within the Dementia Early-Diagnosis Programme at Hospital Universitario San Pedro (Logroño, La Rioja, Spain), using an integrative framework that combined standardised neuropsychological assessment (NEURONORMA project), cerebrospinal fluid biomarkers and magnetic resonance imaging. Cognitively healthy controls underwent the same comprehensive evaluations, with cognitive status re-assessed three years after inclusion, which strengthens diagnostic precision and reduces misclassification. Adjustment of cognitive measures for age and educational level further improves comparability across participants.

Second, the study employed a multidimensional assessment of lifestyle factors, including diet, physical activity, social engagement and leisure activities, with careful adjustment for key confounders. This holistic approach supports the identification of modifiable factors during the preclinical stage of AD, a crucial period for preventive strategies.

Third, the integration of self-reported dietary intake with urinary metabolomic profiling represents a notable methodological advance. The use of objective biomarkers of plant-food and polyphenol intake enhances the reliability of dietary exposure assessment, mitigates recall bias inherent to questionnaires and food records, and enables stratification by metabolic response. Such metabolomic markers align with emerging precision nutrition frameworks and may facilitate personalised dietary recommendations and monitoring tools in at-risk populations.

Despite these strengths, the study has limitations. The relatively small sample size may limit statistical power and the generalisability of findings, particularly for subgroup analyses of individual metabolites and cognitive outcomes. Replication in larger and more diverse populations is therefore warranted. Additionally, although urinary metabolites reflect recent intake and metabolic processing, they may not capture long-term dietary exposures or tissue-specific effects relevant to neurodegenerative processes. Finally, while this cross-sectional analysis suggests associations between diet, metabolite profiles and cognition, causal inferences cannot be established. The ongoing longitudinal follow-up of the cohort will be critical for validating identified biomarkers, assess their predictive value for cognitive decline, and clarify their mechanistic roles in neuroprotection. Future studies incorporating repeated metabolomic measures and expanded dietary assessment will further strengthen evidence for actionable lifestyle interventions.

### Conclusions

In conclusion, our findings support the notion that dietary patterns characterised by higher consumption of vegetables and nuts and lower intake of processed foods, consistent with the MIND diet, are associated with better cognitive outcomes and may represent modifiable factors relevant to the prevention or delay of AD. When combined with cognitively stimulating activities, such as regular reading, these lifestyle factors appear to contribute to a multifactorial framework that supports cognitive resilience and healthy aging during the preclinical stage of AD.

This study further demonstrates the feasibility and added value of LC–MS/MS–based targeted metabolomics for the selective quantification of urinary polyphenol metabolites as objective biomarkers of plant-based food intake. Higher consumption of polyphenol-rich foods was associated with increased urinary concentrations of their corresponding metabolites, including phase-II hepatic conjugates (glucuronides and sulphates), valerolactones, resveratrol derivatives, and a wide range of low-molecular-weight phenolic acids likely derived from gut microbial metabolism. These metabolite profiles provide objective support for self-reported dietary data and may enhance the accuracy of dietary exposure assessment in studies investigating diet-brain health relationships.

Overall, the observed associations between dietary patterns, mental activities, urinary polyphenol metabolites and cognitive performance underscore the potential of integrated lifestyle-based strategies to mitigate cognitive decline in individuals at risk of AD. Although causal relationships cannot be established, these findings reinforce the relevance of diet-microbiota-brain interactions as modifiable determinants of cognitive health. Well-designed longitudinal studies and randomised controlled trials are now warranted to confirm these associations, clarify underlying mechanisms, and evaluate the effectiveness of targeted lifestyle interventions in delaying cognitive symptom onset and improving quality of life in at-risk populations.

## Supplementary Information

Below is the link to the electronic supplementary material.

Supplementary Material 1

Supplementary Material 2

Supplementary Material 3

Supplementary Material 4

Supplementary Material 5

## Data Availability

Additional Supporting Information is available in Supporting Information Tables and Supporting Information Figures. Further data from this study are available on request from the corresponding author upon reasonable request.
