# Supplementary material for: Exploring the associations between lifestyle and dietary patterns with preclinical alzheimer’s disease: findings from La Rioja cohort study
Source: Eur J Nutr. 2026 Jun 6;65(4):156. doi: 10.1007/s00394-026-04011-w (PMC13242501; doi:10.1007/s00394-026-04011-w)
Supplement: Supplementary file 1 — Supplementary Material 1 [file 394_2026_4011_MOESM1_ESM.docx]

**Supplementary Material**

**Supplementary Material Questionnaire S1. LEISURE AND SOCIAL HABITS**

**PARTICIPANT IDENTIFICATION (Code):**

1. **How many hours do you dedicate to watching TV every day? (not including weekends)?**

| 1 hour or less | Between 1-3 hours | Between 3-5 hours | More than 5 hours |
| --- | --- | --- | --- |
|  |  |  |  |

1. **How many hours do you dedicate to reading every day??**

| 1 hour or less | Between 1-3 hours | Between 3-5 hours | More than 5 hours |
| --- | --- | --- | --- |
|  |  |  |  |

1. **How many hours do you dedicate to memory activities (crossword puzzles, word searches, card games, board games...) every day?**

| 1 hour or less | Between 1-3 hours | Between 3-5 hours | More than 5 hours |
| --- | --- | --- | --- |
|  |  |  |  |

1. **How many hours do you dedicate to leisure with friends or family?**

| Every day of the week | Weekends | Weekdays |
| --- | --- | --- |
|  |  |  |

1. **Do you usually eat with company (family, friends, co-workers)?**

Yes □ No □

1. **Do you have hearing problems? Do you use a hearing aid?**

Yes □ No □

**Supplementary Material Questionnaire S2. PHYSICAL ACTIVITY**

**PARTICIPANT IDENTIFICATION (Code):**

**Rapid Classifier of Physical Activity (ClassAF); adapted from the PEFS Guide (Generalitat de Catalunya).**

Put an "X" in the appropriate box:

| **Physical activity at work or at home (L)** | | | |
| --- | --- | --- | --- |
| **0** | **NONE** | Sitting most of the day Mostly sitting throughout the day |  |
| **1** | **LIGHT** | Standing for most of the day and not moving about |  |
| **2** | **MODERATE** | Frequent walking or moving |  |
| **3** | **INTENSE** | Activity requiring intense physical activity |  |
|  |  |  |  |
| **Physical exercise and/or sport (D)** | | | |
| **0** | **NONE** | No exercise of any kind |  |
| **1** | **LIGHT** | Walking, petanque, yoga, ... |  |
| **2** | **MODERATE** | Cycling, gymnastics, aerobics, jogging, tennis, swimming, ... |  |
| **3** | **INTENSE** | Squash, football, basketball, hockey, ... |  |
|  |  |  |  |
| **Weekly Frequency of Physical Exercise and/or Sport (FR)** | | | |
| **0** |  | Never |  |
| **1** |  | Once a week |  |
| **2** |  | Twice a week |  |
| **3** |  | Three or more times a week |  |
| **4** |  | Daily |  |
|  |  | **TOTAL SCORE:** |  |

**Supplementary Material Questionnaire S3**

**FOOD FREQUENCY QUESTIONNARIE (FFQ)**

**PARTICIPANT IDENTIFICATION (Code):**

For each food listed, tick the box indicating how often on average you have used the amount specified during the past year.

|  | AVERAGE CONSUMPTION DURING THE PAST YEAR | | | | | | | | |
| --- | --- | --- | --- | --- | --- | --- | --- | --- | --- |
| **I – DAIRY FOODS** | Never, or less than once per month | PER MONTH | PER WEEK | | | PER DAY | | | |
|  |  | 1-3 | 1 | 2-4 | 5-6 | 1 | 2-3 | 4-6 | 6+ |
| 1. Whole milk (1 cup, 200 cc) |  |  |  |  |  |  |  |  |  |
| 2. Semi-skim milk (1 cup, 200 cc) |  |  |  |  |  |  |  |  |  |
| 3. Skim milk (1 cup, 200 cc) |  |  |  |  |  |  |  |  |  |
| 4. Cream or milk cream (1/2 cup) |  |  |  |  |  |  |  |  |  |
| 5. Soy milk (1 cup, 200cc) |  |  |  |  |  |  |  |  |  |
| 6. Whole yogurt (plain and flavoured) (1, 125 g) |  |  |  |  |  |  |  |  |  |
| 7. Non-fat yogurt (plain and flavoured) (1, 125 g) |  |  |  |  |  |  |  |  |  |
| 8. Cottage cheese or curd (1/2 cup) |  |  |  |  |  |  |  |  |  |
| 9. Portioned or cream cheese (1 portion, 25 g) |  |  |  |  |  |  |  |  |  |
| 10. Other cheeses: cured, semi-cured (Manchego, Bola, Emmental...) (50 g) |  |  |  |  |  |  |  |  |  |
| 11. White or fresh cheese (Burgos, goat…) (50 g) |  |  |  |  |  |  |  |  |  |
| 12. Custard, flan, pudding, chunky yogurt (1, 130 cc) |  |  |  |  |  |  |  |  |  |
| 13. Ice cream (1 cone)  *Ask if it only consumes in summer* |  |  |  |  |  |  |  |  |  |

**Supplementary Material S3. FOOD FREQUENCY QUESTIONNARIE (FFQ) (Continuation)**

|  | AVERAGE CONSUMPTION DURING THE PAST YEAR | | | | | | | | |
| --- | --- | --- | --- | --- | --- | --- | --- | --- | --- |
| **II- EGGS, MEAT, FISH**  (A portion of 100-150 g,  except where otherwise stated) | Never, or less than once per month | PER MONTH | PER WEEK | | | PER DAY | | | |
|  |  | 1-3 | 1 | 2-4 | 5-6 | 1 | 2-3 | 4-6 | 6+ |
| 14. Chicken eggs (one) |  |  |  |  |  |  |  |  |  |
| 15. Chicken or turkey with skin (1 serving or piece) |  |  |  |  |  |  |  |  |  |
| 16. Chicken or turkey without skin (1 serving or piece) |  |  |  |  |  |  |  |  |  |
| 17. Red meat: beef, veal, pork, lamb, rabbit (1 serving)  *Ask which meat is the most frequently consumed* |  |  |  |  |  |  |  |  |  |
| 18. Offal (liver, brains, kidneys…) (1 serving) |  |  |  |  |  |  |  |  |  |
| 19. Cured ham (1 slice, 30 g) |  |  |  |  |  |  |  |  |  |
| 20. Cooked ham, York ham (1 slice, 30 g) |  |  |  |  |  |  |  |  |  |
| 21. Cold cuts (1 slice, 30 g) or other processed meats: salchichón, chorizo, black pudding, mortadella, sausages, butifarra, sobrasada, bacon… (50 g)  *Ask which product is the most frequently consumed* |  |  |  |  |  |  |  |  |  |
| 22. White fish: grouper, sole, sea bream, hake, whiting, cod… (1 dish, piece or serving, 130 g)  *Ask which white fish is the most frequently consumed* |  |  |  |  |  |  |  |  |  |
| 23. Blue fish: sardines, tuna, bonito, mackerel, salmon (1 dish, piece or serving, 130 g)  *Ask which blue fish is the most frequently consumed* |  |  |  |  |  |  |  |  |  |
| 24. Shellfish: clams, mussels and similar (6 pieces), squid, octopus, cuttlefish or crustaceans (1 serving, 200 g) |  |  |  |  |  |  |  |  |  |
| 25. Canned fish and shellfish plain or in oil (sardines, anchovies, bonito, tuna) (1 small can or half a normal can, 50 g) |  |  |  |  |  |  |  |  |  |

**Supplementary Material S3. FOOD FREQUENCY QUESTIONNARIE (FFQ) (Continuation)**

|  | AVERAGE CONSUMPTION DURING THE PAST YEAR | | | | | | | | |
| --- | --- | --- | --- | --- | --- | --- | --- | --- | --- |
| **III - VEGETABLES**  (A portion of 200 g,  except where otherwise stated) | Never, or less than once per month | PER MONTH | PER WEEK | | | PER DAY | | | |
|  |  | 1-3 | 1 | 2-4 | 5-6 | 1 | 2-3 | 4-6 | 6+ |
| 26. Swiss chard, spinach (green leafy vegetables) |  |  |  |  |  |  |  |  |  |
| 27. Cabbage, cauliflower, broccoli |  |  |  |  |  |  |  |  |  |
| 28. Lettuce, endive, escarole (100g) |  |  |  |  |  |  |  |  |  |
| 29. Raw tomato (1, 150 g) |  |  |  |  |  |  |  |  |  |
| 30. Carrot, pumpkin (100 g) |  |  |  |  |  |  |  |  |  |
| 31. Green beans |  |  |  |  |  |  |  |  |  |
| 32. Aubergine, courgette, cucumber |  |  |  |  |  |  |  |  |  |
| 33. Artichoke (100 g) |  |  |  |  |  |  |  |  |  |
| 34. Red or green peppers (150 g) |  |  |  |  |  |  |  |  |  |
| 35. Asparagus  *Ask if it only consumes in season* |  |  |  |  |  |  |  |  |  |
| 36. Purple cabbage  *Ask if it only consumes in season* |  |  |  |  |  |  |  |  |  |
| 37. Celery (100 g) |  |  |  |  |  |  |  |  |  |
| 38. Leek (100 g) |  |  |  |  |  |  |  |  |  |
| 39. Spanish soupe “gazpacho” (1 glass, 200 g)  *Ask if it only consumes in summer* |  |  |  |  |  |  |  |  |  |
| 40. Onion (half an onion, 50 g) *(if usually consumed fresh)* |  |  |  |  |  |  |  |  |  |
| 41. Garlic (1 clove) |  |  |  |  |  |  |  |  |  |
| 42. Parsley, thyme, bay leaf, oregano... (a little)  *Ask if they use them regularly for cooking* |  |  |  |  |  |  |  |  |  |
| 43. Home fries (1 serving, 150 g) |  |  |  |  |  |  |  |  |  |
| 44. Commercial potato chips (1 bag, 50 g) |  |  |  |  |  |  |  |  |  |
| 45. Roast or boiled potatoes |  |  |  |  |  |  |  |  |  |
| 46. Mushrooms  *Ask if it only consumes in season* |  |  |  |  |  |  |  |  |  |

**Supplementary Material S3. FOOD FREQUENCY QUESTIONNARIE (FFQ) (Continuation)**

|  | AVERAGE CONSUMPTION DURING THE PAST YEAR | | | | | | | | |
| --- | --- | --- | --- | --- | --- | --- | --- | --- | --- |
| **IV- FRUITS**  * indicate if it is seasonal consumption | Never, or less than once per month | PER MONTH | PER WEEK | | | PER DAY | | | |
|  |  | 1-3 | 1 | 2-4 | 5-6 | 1 | 2-3 | 4-6 | 6+ |
| 47. Kiwi (one, 100 g) |  |  |  |  |  |  |  |  |  |
| 48. Citrus fruits: orange (one), tangerines (two)  *Ask if it only consumes in season* |  |  |  |  |  |  |  |  |  |
| 49. Banana (one) |  |  |  |  |  |  |  |  |  |
| 50. Apple or pear (one) |  |  |  |  |  |  |  |  |  |
| 51. Strawberries (6 pieces, 1 dessert dish)  *Ask if it only consumes in season* |  |  |  |  |  |  |  |  |  |
| 52. Raspberries, blueberries, blackberries (1 cup, 100 g) |  |  |  |  |  |  |  |  |  |
| 53. Cherries, plums (1 cup, 1 dessert dish, 100 g)  *Ask if it only consumes in season* |  |  |  |  |  |  |  |  |  |
| 54. Apricot, peach, nectarine (one piece)  *Ask if it only consumes in season* |  |  |  |  |  |  |  |  |  |
| 55. Watermelon, melon (1 slice, 200-250 g)  *Ask if it only consumes in season* |  |  |  |  |  |  |  |  |  |
| 56. Grapes (one bunch, 1 dessert dish)  *Ask if it only consumes in season* |  |  |  |  |  |  |  |  |  |
| 57. Fruits in syrup or in their juice (2 units) |  |  |  |  |  |  |  |  |  |
| 58. Dates, dried figs, raisins, prunes (150 g) |  |  |  |  |  |  |  |  |  |
| 59. How many days a week do you eat fruit for dessert?  *Write down a number:* |  |  |  |  |  |  |  |  |  |

**Supplementary Material S3. FOOD FREQUENCY QUESTIONNARIE (FFQ) (Continuation)**

|  | AVERAGE CONSUMPTION DURING THE PAST YEAR | | | | | | | | |
| --- | --- | --- | --- | --- | --- | --- | --- | --- | --- |
| **V- LEGUMES AND CEREALS**  One dish or serving (150 g) | Never, or less than once per month | PER MONTH | PER WEEK | | | PER DAY | | | |
|  |  | 1-3 | 1 | 2-4 | 5-6 | 1 | 2-3 | 4-6 | 6+ |
| 60. Lentils, beans, chickpeas  (1 dish, 150 g cooked) |  |  |  |  |  |  |  |  |  |
| 61. Peas, broad beans (1 dish, 150 g cooked) |  |  |  |  |  |  |  |  |  |
| 62. White bread (fresh or sliced)  (3 slices, 75 g) |  |  |  |  |  |  |  |  |  |
| 63. Wholemeal bread (3 slices, 75 g) or wholegrain cereals (rice, pasta) |  |  |  |  |  |  |  |  |  |
| 64. White rice (60 g raw) |  |  |  |  |  |  |  |  |  |
| 65. Refined pasta: noodles, macaroni, spaghetti… (60 g raw) |  |  |  |  |  |  |  |  |  |
| 66. Wholegrain breakfast cereals: oat flakes, all-bran… (30 g) |  |  |  |  |  |  |  |  |  |

|  | AVERAGE CONSUMPTION DURING THE PAST YEAR | | | | | | | | |
| --- | --- | --- | --- | --- | --- | --- | --- | --- | --- |
| **VI- OILS AND FATS**  One tablespoon or individual portion.  For frying, spreading, dipping in bread, for dressing, or for salads, you use in total: | Never, or less than once per month | PER MONTH | PER WEEK | | | PER DAY | | | |
|  |  | 1-3 | 1 | 2-4 | 5-6 | 1 | 2-3 | 4-6 | 6+ |
| 67. Olive oil (one tablespoon) |  |  |  |  |  |  |  |  |  |
| 68. Extra virgin olive oil (one tablespoon) |  |  |  |  |  |  |  |  |  |
| 69. Pomace olive oil (one tablespoon) |  |  |  |  |  |  |  |  |  |
| 70. Corn, sunflower, soybean or other oil (one tablespoon) |  |  |  |  |  |  |  |  |  |
| 71. Margarine (single portion, 12 g) |  |  |  |  |  |  |  |  |  |
| 72. Butter (single portion, 12 g) |  |  |  |  |  |  |  |  |  |
| 73. Lard (10 g) |  |  |  |  |  |  |  |  |  |
| 74. Almonds, peanuts, hazelnuts, pistachios (30 g) |  |  |  |  |  |  |  |  |  |
| 75. Walnuts (30 g) |  |  |  |  |  |  |  |  |  |
| 76. Olives (10 units) |  |  |  |  |  |  |  |  |  |

**Supplementary Material S3. FOOD FREQUENCY QUESTIONNARIE (FFQ) (Continuation)**

|  | AVERAGE CONSUMPTION DURING THE PAST YEAR | | | | | | | | |
| --- | --- | --- | --- | --- | --- | --- | --- | --- | --- |
| **VII-SWEET PASTRIES AND CONFECTIONERY** | Never, or less than once per month | PER MONTH | PER WEEK | | | PER DAY | | | |
|  |  | 1-3 | 1 | 2-4 | 5-6 | 1 | 2-3 | 4-6 | 6+ |
| 77. Maria type cookies (4-6 units, 50 g) |  |  |  |  |  |  |  |  |  |
| 78. Wholemeal or fibre biscuits (4-6 pieces, 50 g) |  |  |  |  |  |  |  |  |  |
| 79. Chocolate biscuits (4 pieces, 50 g) |  |  |  |  |  |  |  |  |  |
| 80. Homemade pastries and biscuits (50 g) |  |  |  |  |  |  |  |  |  |
| 81. Industrial pastries: croissants, muffins, churros… (one, 1 serving) |  |  |  |  |  |  |  |  |  |
| 82. Chocolate (30g)  *Annotate type* |  |  |  |  |  |  |  |  |  |
| 83. Cocoa powder, cocoa soluble (1 dessert spoon) |  |  |  |  |  |  |  |  |  |
| 84. Nougat (1/8 bar, 40 g)  *Ask if it only consumes in Christmas* |  |  |  |  |  |  |  |  |  |
| 85. Mantecados, mazapan (90 g)  *Ask if it only consumes in Christmas* |  |  |  |  |  |  |  |  |  |

|  | AVERAGE CONSUMPTION DURING THE PAST YEAR | | | | | | | | |
| --- | --- | --- | --- | --- | --- | --- | --- | --- | --- |
| **VIII-MISCELLANEOUS** | Never, or less than once per month | PER MONTH | PER WEEK | | | PER DAY | | | |
|  |  | 1-3 | 1 | 2-4 | 5-6 | 1 | 2-3 | 4-6 | 6+ |
| 86. Croquettes, dumplings, pre-cooked/fried food (one) |  |  |  |  |  |  |  |  |  |
| 87. Soups and creams in sachets (1 dish) |  |  |  |  |  |  |  |  |  |
| 88. Mustard (one tablespoon) |  |  |  |  |  |  |  |  |  |
| 89. Commercial mayonnaise (1 tablespoon, 20 g) |  |  |  |  |  |  |  |  |  |
| 90. Fried tomato sauce, ketchup  (1 tablespoon) |  |  |  |  |  |  |  |  |  |
| 91. Spicy: tabasco, pepper, paprika (a little)  *Ask if it is used as a regular condiment* |  |  |  |  |  |  |  |  |  |
| 92. Salt (a little)  *Ask whether it is regularly added to meals* |  |  |  |  |  |  |  |  |  |
| 93. Jams (1 tablespoon, 10 g) |  |  |  |  |  |  |  |  |  |
| 94. Sugar, honey (1 tablespoon, 6 g) |  |  |  |  |  |  |  |  |  |
| 95. Snacks other than potato crisps: popcorn, corn... (1 bag, 50 g) |  |  |  |  |  |  |  |  |  |
| 96. Other frequently consumed foods (specify): | | | | | | | | | |
|  |  |  |  |  |  |  |  |  |  |
|  |  |  |  |  |  |  |  |  |  |
|  |  |  |  |  |  |  |  |  |  |
|  |  |  |  |  |  |  |  |  |  |

**Supplementary Material S3. FOOD FREQUENCY QUESTIONNARIE (FFQ) (Continuation)**

|  | AVERAGE CONSUMPTION DURING THE PAST YEAR | | | | | | | | |
| --- | --- | --- | --- | --- | --- | --- | --- | --- | --- |
| **IX-BEVERAGES** | Never, or less than once per month | PER MONTH | PER WEEK | | | PER DAY | | | |
|  |  | 1-3 | 1 | 2-4 | 5-6 | 1 | 2-3 | 4-6 | 6+ |
| 97. Water (1 glass, 200cc) |  |  |  |  |  |  |  |  |  |
| 98. Carbonated drinks with sugar: cola, lemonade, tonic, soda... (1 bottle, 200 cc) |  |  |  |  |  |  |  |  |  |
| 99. Low-calorie carbonated drinks, light drinks (1 bottle, 200 cc) |  |  |  |  |  |  |  |  |  |
| 100. Natural orange juice (1 glass, 200 cc) |  |  |  |  |  |  |  |  |  |
| 101. Commercial fruit juices (200 cc) |  |  |  |  |  |  |  |  |  |
| 102. Vegetable drinks: rice, oatmeal, almond... (1 glass, 200cc) |  |  |  |  |  |  |  |  |  |
| 103. Decaffeinated coffee (1 cup, 50 cc) |  |  |  |  |  |  |  |  |  |
| 104. Coffee (1 cup, 50 cc) |  |  |  |  |  |  |  |  |  |
| 105. Tea (1 cup, 50 cc) |  |  |  |  |  |  |  |  |  |
| 106. Must (100 cc) |  |  |  |  |  |  |  |  |  |
| 107. Muscatel wine (50 cc) |  |  |  |  |  |  |  |  |  |
| 108. Rosé wine (100 cc) |  |  |  |  |  |  |  |  |  |
| 109. Young red wine, crianza red wine (100 cc) |  |  |  |  |  |  |  |  |  |
| 110. Reserve red wine (100 cc) |  |  |  |  |  |  |  |  |  |
| 111. White wine (100 cc) |  |  |  |  |  |  |  |  |  |
| 112. Cava (100 cc) |  |  |  |  |  |  |  |  |  |
| 113. Beer (1 can, 330 cc) |  |  |  |  |  |  |  |  |  |
| 114. Liqueurs (1 glass, 50 cc) |  |  |  |  |  |  |  |  |  |
| 115. Distilled spirits: whisky, vodka, gin, cognac... (1 glass, 50 cc) |  |  |  |  |  |  |  |  |  |

At what age did you start drinking alcohol (wine, beer or spirits), including with meals on a regular basis (more than seven "drinks" a week)?____________________________________________________

How many years have you been drinking alcohol regularly (more than seven "drinks" a week)?________

If you have taken any vitamins and/or minerals (including calcium) or special dietary products in the past year, please indicate the brand and frequency of use:

|  | Never, or less than once per month | PER MONTH | PER WEEK | | | PER DAY | | | |
| --- | --- | --- | --- | --- | --- | --- | --- | --- | --- |
|  |  | 1-3 | 1 | 2-4 | 5-6 | 1 | 2-3 | 4-6 | 6+ |
|  |  |  |  |  |  |  |  |  |  |
|  |  |  |  |  |  |  |  |  |  |

**Supplementary Material Table S4.** The 15-point MIND diet Score (partially based on the Mediterranean and DASH diets Adherence Screener) based on some questions of the Food Frequency Questionnaire (FFQ)(Morris et al., 2015).

|  | 0 | 0.5 | 1 |
| --- | --- | --- | --- |
| Green Leafy Vegetables^a^ | ≤ 2 servings/wk | > 2 a < 6/wk | ≥ 6 servings/wk |
| Other Vegetables^b^ | < 5 serving/wk | 5 - < 7 servings/wk | ≥ 1 serving/day |
| Berries^c^ | ≤ 1 serving/wk | 1 serving/wk | ≥ 2 serving/wk |
| Nuts^d^ | ≤ 1 serving/month | 1/month - < 5 wk | ≥ 5 serving/wk |
| Olive oil | Not primary oil |  | Primary oil used |
| Whole Grains | < 1 serving/day | 1-2 serving/day | ≥ 3 serving/day |
| Fish (not fried)^e^ | Rarely | 1-3 serving/month | ≥ 1 servings/wk |
| Beans^f^ | < 1 serving/wk | 1-3 serving/wk | > 2 serving/wk |
| Poultry (not fried)^g^ | < 1 serving/wk | 1 serving/wk | ≥ 2 serving/wk |
| Butter, Margarine | > 2 tablespoon/day | 1-2 tablespoon/day | < 1 tablespoon/day |
| Cheese | +7 serving/wk | 1-6 serving/wk | < 1 serving/wk |
| Red Meat and products^h^ | +7 serving/wk | 4-6 serving/wk | < 4 serving/wk |
| Fast Fried Foods^i^ | +4 serving/wk | 1-3 serving/wk | < 1 serving/wk |
| Pastries & Sweets^j^ | +7 serving/wk | 5-6 serving/wk | < 1 serving/wk |
| Wine | > 2 glass/day or never | 1/month – 6/wk | 1 glass/day |
| TOTAL SCORE |  |  | 15 |

*^a^ cabbage,^,^ kale, collards, greens; spinach; lettuce/tossed salad*

*^b^ green/red peppers, squash, cooked carrots, raw carrots, broccoli, celery, potatoes, peas or lima beans, potatoes, tomatoes, tomato sauce, string beans, beets, corn, zucchini/summer squash/eggplant, coleslaw, potato salad*

*^c^ strawberries*

*^d^* *walnuts, pistachios, hazelnuts, almonds, peanuts*

*^e^ tuna sandwich, fresh fish as main dish; not fried fish cakes, sticks, or sandwiches*

*^f^ beans, lentils, soybeans*

*^g^ chicken or turkey sandwich, chicken or turkey as main dish and never eat fried at home or away from home*

*^h^ cheeseburger, hamburger, beef tacos/burritos, hot dogs/sausages, roast beef or ham sandwich, salami, bologna, or other deli meat sandwich, beef (steak, roast) or lamb as main dish, pork or ham as main dish, meatballs or meatloaf*

*^i^ How often do you eat fried food away from home (like French fries, chicken nuggets)?*

*^j^ biscuit/roll, poptarts, cake, snack cakes/twinkies, Danish/sweetrolls/pastry, donuts, cookies, brownies, pie, candy bars, other candy, ice cream, pudding, milkshakes/frappes*

**Supplementary Material Table S5.** The 14-point MEDAS Index (Mediterranean Diet Adherence Screener) based on some questions of the Food Frequency Questionnaire (FFQ)(Martinez-González et al., 2012).

|  | **Frequency**  **(Criterion to score 1 point. Otherwise, 0 recorded)** |
| --- | --- |
| 1. Do you use olive oil as the principal source of fat for cooking? | Yes |
| 2. How much olive oil do you consume per day (including that used in frying, salads, meals eaten away from home, etc.)? | ≥ 4 Tbsp (tablespoon = 13.5 g) |
| 3. How many servings of vegetables do you consume per day? A full serving is 200 g. | ≥ 2 |
| 4. How many pieces of fruit (including fresh-squeezed juice) do you consume per day? | ≥ 3 |
| 5. How many servings of red meat, hamburger, or sausages do you consume per day? A full serving is 100–150 g. | < 1 |
| 6. How many servings (12 g) of butter, margarine, or cream do you consume per day? | < 1 |
| 7. How many carbonated and/or sugar-sweetened beverages do you consume per day? | < 1 |
| 8. Do you drink wine? How much do you consume per week? | ≥ 7 cups (1 cup= 100 mL) |
| 9. How many servings (150 g) of pulses do you consume per week? | ≥ 3 |
| 10. How many servings of fish/seafood do you consume per week? (100–150 g of fish, 4–5 pieces or 200 g of seafood) | ≥ 3 |
| 11. How many times do you consume commercial (not homemade) pastry such as cookies or cake per week? | < 2 |
| 12. How many times do you consume nuts per week? (1 serving = 30 g) | ≥ 3 |
| 13. Do you prefer to eat chicken, turkey or rabbit instead of beef, pork, hamburgers, or sausages? | Yes |
| 14. How many times per week do you consume boiled vegetables, pasta, rice, or other dishes with a sauce of tomato, garlic, onion, or leeks sautéed in olive oil? | ≥ 2 |
| TOTAL SCORE | 14 |

**Supplementary Material Questionnaire S6. THREE-DAY FOOD RECORD (3-DFR)**

**PARTICIPANT IDENTIFICATION (Code):**

**INSTRUCTIONS TO COMPLETE THE QUESTIONNAIRE**

As part of your collaboration in this study, we need to know about your eating habits. To this, we ask you to write down on the following pages exactly what you consume during the 3 days indicated.

- We are interested in your regular consumption, so please **try to avoid any changes in your eating habits**.
- Each time you eat food, you should **specify as precisely as possible the type of food eaten**. For example, you would have to specify:
- Milk: whole, skimmed or semi-skimmed.
- Yogurt: whole or skimmed, natural or with fruit or flavours, sweetened or enriched…
- Cheese: name of the cheese and its fat content (if known).
- Meat or fish: the type of fish or meat (tuna, sole, hake or veal, chicken, lamb...), specifying whether it is a loin, leg, breast, ribs…
- Vegetables and cereals: the type of vegetable or cereal, and whether it is fresh, frozen or canned food.
- Bread: white or wholemeal bread.
- Fats and oils: butter, margarine, light margarine, cream, oil (specify the type of oil: olive oil, sunflower oil, corn oil...).
- Ready meals, cakes and biscuits: the name of the food. You can provide us with the nutritional composition or the list of ingredients described on the packaging.
- Fruit: the name of the fruit and whether it is fresh or preserved.
- Beverages: the type of drink (whether it is light or not, with or without caffeine, natural or bottled juices, alcohol content if necessary...).
- You must **specify the quantity consumed of each food**, taking as a reference:
- The weight specified on the package or packaging, or as measured by yourself.
- The number of tablespoons.
- Number of glasses, cups or bowls, after checking their capacity.
- The number of units or parts of units (e.g. one packet of biscuits, half a packet, 5 biscuits...).
- You should indicate the consumption of any food or drink consumed at and between meals (sweets, chocolate, cakes, biscuits, juices, coffee…).
- For each meal, the cooking method and the type of fat used for cooking (olive oil, sunflower oil, margarine or butter...) must be specified.
- Do not forget to indicate the amount of sugar or honey added to yogurt, coffee, tea or herbal teas.

**THANK YOU VERY MUCH FOR YOUR COOPERATION**

**Supplementary Material S6. THREE-DAY FOOD RECORD (3-DFR) (Continuation)**

1

Day 1

**Date: ________________**

**Day of the week:**

- **Monday**
- **Tuesday**
- **Wednesday**
- **Thursday**
- **Friday**
- **Saturday**
- **Sunday**

**Date: ____________**

**Breakfast**

| **FOODS** | QUANTITY |
| --- | --- |
|  |  |
| *Coffee, tea, milk, juices...* |  |

**Mid-morning**

| **FOODS** | QUANTITY |
| --- | --- |
|  |  |
| *Coffee, tea, milk, juices...* |  |

**Lunch**

| **Remenber to specify:** | **FOODS** | QUANTITY | **PREPARATION**  **OR DRESSING** | *QUANTITY*DRESSING |
| --- | --- | --- | --- | --- |
| **Appetizer:** *drink, crackers …* | | | | |
| **First dish:** *vegetables, cereals, potatoes, legumes …* | | | | |
| **Second dish:** *meat, fish, eggs …* | | | | |
| **Side dish:** *vegetables, mushrooms, potatoes, rice …* | | | | |
| **Dessert:** *fruit, yogurt, cake, ice cream* … | | | | |
| **Bread:** *white bread, wholemeal bread …* | | | | |
| **Beverages:** *water, fruit juices, beer, wine, coffee, tea, coffee, tea …* | | | | |

**Date: ____________**

**Afternoon snack**

| **FOODS** | QUANTITY |
| --- | --- |
| *Coffee, tea, milk, juices...* |  |

**Dinner**

| **Remenber to specify:** | **FOODS** | QUANTITY | **PREPARATION**  **OR DRESSING** | *QUANTITY*DRESSING |
| --- | --- | --- | --- | --- |
| **Appetizer:** *drink, crackers …* | | | | |
| **First dish:** *vegetables, cereals, potatoes, legumes …* | | | | |
| **Second dish:** *meat, fish, eggs …* | | | | |
| **Side dish:** *vegetables, mushrooms, potatoes, rice …* | | | | |
| **Dessert:** *fruit, yogurt, cake, ice cream* … | | | | |
| **Bread:** *white bread, wholemeal bread …* | | | | |
| **Beverages:** *water, fruit juices, beer, wine, coffee, tea, coffee, tea …* | | | | |

**Others**

| **FOODS** | QUANTITY |
| --- | --- |
|  |  |
| *Coffee, tea, milk, juices...* |  |
|  |  |

**Supplementary Material S6. THREE-DAY FOOD RECORD (3-DFR) (Continuation)**

Day 2

**Date: ________________**

**Day of the week:**

- **Monday**
- **Tuesday**
- **Wednesday**
- **Thursday**
- **Friday**
- **Saturday**
- **Sunday**

**Date: ____________**

**Breakfast**

| **FOODS** | QUANTITY |
| --- | --- |
|  |  |
| *Coffee, tea, milk, juices...* |  |

**Mid-morning**

| **FOODS** | QUANTITY |
| --- | --- |
|  |  |
| *Coffee, tea, milk, juices...* |  |

**Lunch**

| **Remenber to specify:** | **FOODS** | QUANTITY | **PREPARATION**  **OR DRESSING** | *QUANTITY*DRESSING |
| --- | --- | --- | --- | --- |
| **Appetizer:** *drink, crackers …* | | | | |
| **First dish:** *vegetables, cereals, potatoes, legumes …* | | | | |
| **Second dish:** *meat, fish, eggs …* | | | | |
| **Side dish:** *vegetables, mushrooms, potatoes, rice …* | | | | |
| **Dessert:** *fruit, yogurt, cake, ice cream* … | | | | |
| **Bread:** *white bread, wholemeal bread …* | | | | |
| **Beverages:** *water, fruit juices, beer, wine, coffee, tea, coffee, tea …* | | | | |

**Afternoon snack**

| **FOODS** | QUANTITY |
| --- | --- |
| *Coffee, tea, milk, juices...* |  |

**Date: ____________**

**Dinner**

| **Remenber to specify:** | **FOODS** | QUANTITY | **PREPARATION**  **OR DRESSING** | *QUANTITY*DRESSING |
| --- | --- | --- | --- | --- |
| **Appetizer:** *drink, crackers …* | | | | |
| **First dish:** *vegetables, cereals, potatoes, legumes …* | | | | |
| **Second dish:** *meat, fish, eggs …* | | | | |
| **Side dish:** *vegetables, mushrooms, potatoes, rice …* | | | | |
| **Dessert:** *fruit, yogurt, cake, ice cream* … | | | | |
| **Bread:** *white bread, wholemeal bread …* | | | | |
| **Beverages:** *water, fruit juices, beer, wine, coffee, tea, coffee, tea …* | | | | |

**Others**

| **FOODS** | QUANTITY |
| --- | --- |
|  |  |
| *Coffee, tea, milk, juices...* |  |
|  |  |

**Supplementary Material S6. THREE-DAY FOOD RECORD (3-DFR) (Continuation)**

Day 3

**Date: ________________**

**Day of the week:**

- **Monday**
- **Tuesday**
- **Wednesday**
- **Thursday**
- **Friday**
- **Saturday**
- **Sunday**

**Date: ____________**

**Breakfast**

| **FOODS** | QUANTITY |
| --- | --- |
| *Coffee, tea, milk, juices...* |  |

**Mid-morning**

| **FOODS** | QUANTITY |
| --- | --- |
|  |  |
| *Coffee, tea, milk, juices...* |  |

**Lunch**

| **Remenber to specify:** | **FOODS** | QUANTITY | **PREPARATION**  **OR DRESSING** | *QUANTITY*DRESSING |
| --- | --- | --- | --- | --- |
| **Appetizer:** *drink, crackers …* | | | | |
| **First dish:** *vegetables, cereals, potatoes, legumes …* | | | | |
| **Second dish:** *meat, fish, eggs …* | | | | |
| **Side dish:** *vegetables, mushrooms, potatoes, rice …* | | | | |
| **Dessert:** *fruit, yogurt, cake, ice cream* … | | | | |
| **Bread:** *white bread, wholemeal bread …* | | | | |
| **Beverages:** *water, fruit juices, beer, wine, coffee, tea, coffee, tea …* | | | | |

**Date: ____________**

**Afternoon snack**

| **FOODS** | QUANTITY |
| --- | --- |
| *Coffee, tea, milk, juices...* |  |

**Dinner**

| **Remenber to specify:** | **FOODS** | QUANTITY | **PREPARATION**  **OR DRESSING** | *QUANTITY*DRESSING |
| --- | --- | --- | --- | --- |
| **Appetizer:** *drink, crackers …* | | | | |
| **First dish:** *vegetables, cereals, potatoes, legumes …* | | | | |
| **Second dish:** *meat, fish, eggs …* | | | | |
| **Side dish:** *vegetables, mushrooms, potatoes, rice …* | | | | |
| **Dessert:** *fruit, yogurt, cake, ice cream* … | | | | |
| **Bread:** *white bread, wholemeal bread …* | | | | |
| **Beverages:** *water, fruit juices, beer, wine, coffee, tea, coffee, tea …* | | | | |

**Others**

| **FOODS** | QUANTITY |
| --- | --- |
| *Coffee, tea, milk, juices...* |  |
|  |  |

| **CLARIFICATIONS, RECIPES OR OTHER FOOD INFORMATION** |
| --- |
|  |
