# Supplementary material for: Exploring the associations between lifestyle and dietary patterns with preclinical alzheimer’s disease: findings from La Rioja cohort study
Source: Eur J Nutr. 2026 Jun 6;65(4):156. doi: 10.1007/s00394-026-04011-w (PMC13242501; doi:10.1007/s00394-026-04011-w)
Supplement: Supplementary file 2 — Supplementary Material 2 [file 394_2026_4011_MOESM2_ESM.ppt]

## Slide 1
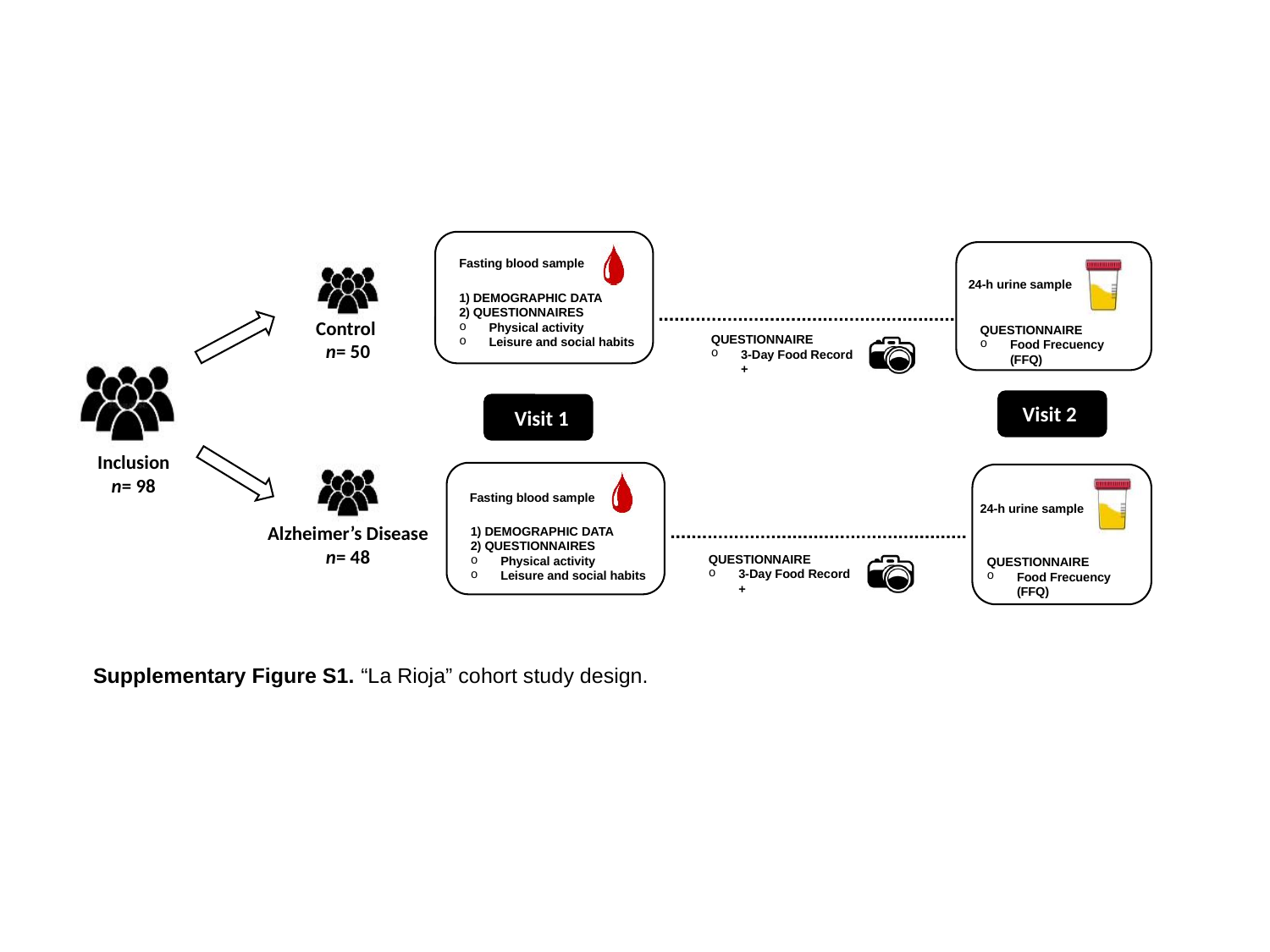

1) DEMOGRAPHIC DATA
2) QUESTIONNAIRES
Physical activity
Leisure and social habits
QUESTIONNAIRE
Food Frecuency (FFQ)
QUESTIONNAIRE
3-Day Food Record +
Visit 2
Visit 1
Fasting blood sample
24-h urine sample
Control
n= 50
Inclusion
n= 98
1) DEMOGRAPHIC DATA
2) QUESTIONNAIRES
Physical activity
Leisure and social habits
QUESTIONNAIRE
Food Frecuency (FFQ)
Fasting blood sample
24-h urine sample
Alzheimer’s Disease
n= 48
QUESTIONNAIRE
3-Day Food Record +
Supplementary Figure S1. “La Rioja” cohort study design.
